# Global Analysis of Photosynthesis Transcriptional Regulatory Networks

**DOI:** 10.1371/journal.pgen.1004837

**Published:** 2014-12-11

**Authors:** Saheed Imam, Daniel R. Noguera, Timothy J. Donohue

**Affiliations:** 1 Program in Cellular and Molecular Biology, University of Wisconsin – Madison, Madison, Wisconsin, United States of America; 2 Department of Bacteriology, University of Wisconsin – Madison, Wisconsin Energy Institute, Madison, Wisconsin, United States of America; 3 DOE Great Lakes Bioenergy Research Center, University of Wisconsin – Madison, Madison, Wisconsin, United States of America; 4 Department of Civil and Environmental Engineering, University of Wisconsin – Madison, Madison, Wisconsin, United States of America; University of Geneva Medical School, Switzerland

## Abstract

Photosynthesis is a crucial biological process that depends on the interplay of many components. This work analyzed the gene targets for 4 transcription factors: FnrL, PrrA, CrpK and MppG (RSP_2888), which are known or predicted to control photosynthesis in *Rhodobacter sphaeroides*. Chromatin immunoprecipitation followed by high-throughput sequencing (ChIP-seq) identified 52 operons under direct control of FnrL, illustrating its regulatory role in photosynthesis, iron homeostasis, nitrogen metabolism and regulation of sRNA synthesis. Using global gene expression analysis combined with ChIP-seq, we mapped the regulons of PrrA, CrpK and MppG. PrrA regulates ∼34 operons encoding mainly photosynthesis and electron transport functions, while CrpK, a previously uncharacterized Crp-family protein, regulates genes involved in photosynthesis and maintenance of iron homeostasis. Furthermore, CrpK and FnrL share similar DNA binding determinants, possibly explaining our observation of the ability of CrpK to partially compensate for the growth defects of a ΔFnrL mutant. We show that the Rrf2 family protein, MppG, plays an important role in photopigment biosynthesis, as part of an incoherent feed-forward loop with PrrA. Our results reveal a previously unrealized, high degree of combinatorial regulation of photosynthetic genes and significant cross-talk between their transcriptional regulators, while illustrating previously unidentified links between photosynthesis and the maintenance of iron homeostasis.

## Author Summary

Photosynthetic organisms are among the most abundant life forms on earth. Their unique ability to harvest solar energy and use it to fix atmospheric carbon dioxide is at the foundation of the global food chain. This paper reports the first comprehensive analysis of networks that control expression of photosynthesis genes using *Rhodobacter sphaeroides*, a microbe that has been studied for decades as a model of solar energy capture and other aspects of the photosynthetic lifestyle. We find a previously unappreciated complexity in the level of control of photosynthetic genes, while identifying new links between photosynthesis and central processes like iron availability. This organism is an ancestor of modern day plants, so our data can inform studies in other photosynthetic organisms and improve our ability to harness solar energy for food and industrial processes.

## Introduction

Photosynthetic organisms are central to life on the planet. Their ability to harness solar energy and fix atmospheric carbon dioxide makes them integral parts of most ecosystems. Furthermore, many photosynthetic microbes, either naturally or via modifications, are capable of producing a variety of valuable commodities such as grain for food, hydrocarbons, hydrogen gas and valuable chemicals [1]–[4]. These properties will likely make them important in efforts to develop more sustainable societies. We are interested in obtaining new knowledge about the transcriptional networks of photosynthetic cells that underlie these important activities.

Anoxygenic photosynthetic bacteria have and continue to provide significant insight into the networks that govern photosynthetic activities because of their ease of growth, genetic tractability, and prior knowledge about solar energy capture and other aspects of this lifestyle [3], [5]. The advent of genomic approaches has allowed development of metabolic and transcriptional regulatory network (TRN) models for bacterial photosynthesis, the latter of which has led to predictions about regulatory networks in photosynthetic cells that extend beyond prior knowledge [6]–[10]. Thus, there is likely still much more to be learned about photosynthesis through testing the predictions of metabolic and TRN models in well-studied photosynthetic organisms.

To obtain this new knowledge, we analyze *Rhodobacter sphaeroides*, the best studied member of the purple non-sulfur bacteria – a group of photosynthetic microbes displaying great metabolic versatility and having significant biotechnological potential [1], [7], [11]–[18]. *R. sphaeroides* is capable of growing by aerobic respiration, anaerobic respiration and anaerobic anoxygenic photosynthesis. Prior analysis indicates that transitions between aerobic respiratory and anaerobic photosynthetic growth is achieved, in part, via a TRN involving 3 global transcription factors (TFs) – PrrA, FnrL and PpsR – that act to activate or repress relevant operons depending on the presence of oxygen or other signals. For instance, PrrA (the response regulator of the PrrAB two component system) and FnrL (the *R. sphaeroides* homolog of FNR) directly activate transcription of photosynthesis related genes at low oxygen tensions [9], [19]–[25]. On the other hand, PpsR represses the expression of photosynthesis related genes at high oxygen tensions [8], [26], [27]. In addition to these TFs, a small non-coding RNA, PcrZ has recently been implicated in the regulation of photosynthesis gene expression in *R. sphaeroides* [28]. While there is considerable information on how these regulators impact some photosynthesis genes, global information on their targets and how they act together to impact this lifestyle is lacking. Furthermore, a large-scale reconstruction of the *R. sphaeroides* TRN [10], which combined comparative genomics analysis with global gene expression data, predicted that two previously uncharacterized TFs, CrpK and RSP_2888 (hereafter referred to as *m*odulator of *p*hoto*p*igment *g*enes, MppG), were involved in controlling the transcription of a number of operons that encode key functions involved in photosynthesis in *R. sphaeroides*, suggesting that the photosynthetic TRN of this organism is more complex than previously thought.

In this work, we use a combination of genetic, genomic and physiological analysis to dissect the roles of 4 TFs known or predicted to be involved in the regulation of the photosynthetic lifestyle of *R. sphaeroides*. The regulons of the previously characterized TFs, PrrA and FnrL, were refined and extended, while those of CrpK and MppG were characterized for the first time. Our analysis confirmed many predictions of the large-scale *R. sphaeroides* TRN, revealed the existence of significant overlap in direct targets for these TFs, as well as the high degree of combinational regulation of key operons. We also identified how components in this photosynthetic TRN provide robustness and fine-tuned expression of target genes. Overall, this study provides a large amount of new insight into the photosynthetic TRN of *R. sphaeroides* that is likely to be conserved in other related photosynthetic bacteria.

## Results

### Genome-wide analysis of known regulators of photosynthesis in *R. sphaeroides*

Based on previous analysis in *R. sphaeroides* and related purple non-sulfur bacteria, FnrL, PrrA and PpsR have been identified as key regulators of the photosynthetic lifestyle [8], [9], [20], [24], [27], [29]. We have previously characterized the genome-wide binding sites of PpsR via chromatin immunoprecipitation followed by sequencing (ChIP-seq) and gene expression analysis [10]. This analysis identified a total of 15 PpsR target operons, of which 13 had photosynthesis related functions. Here, we analyze the regulons of FnrL and PrrA using both ChIP-seq and global gene expression analysis.

### FnrL—a global regulator of anaerobic growth in *R. sphaeroides*

FnrL is an iron-sulfur cluster-containing Crp-family TF that has been reported to be essential for both photosynthetic and anaerobic respiratory growth in *R. sphaeroides* [9], [25]. ChIP-chip analysis has previously been used to map genome-wide FnrL binding sites *in vivo*, identifying targets that indicate the direct involvement of this protein in a host of processes including those required for photosynthetic and anaerobic respiratory growth [24]. However, a large-scale reconstruction of *R. sphaeroides* TRN predicted that the FnrL regulon is significantly larger than previous analyses suggested. Thus, we re-examined the FnrL regulon using new and higher resolution complementary genomic approaches.

#### Analyzing the FnrL regulon using ChIP-seq

**Figure 1:**
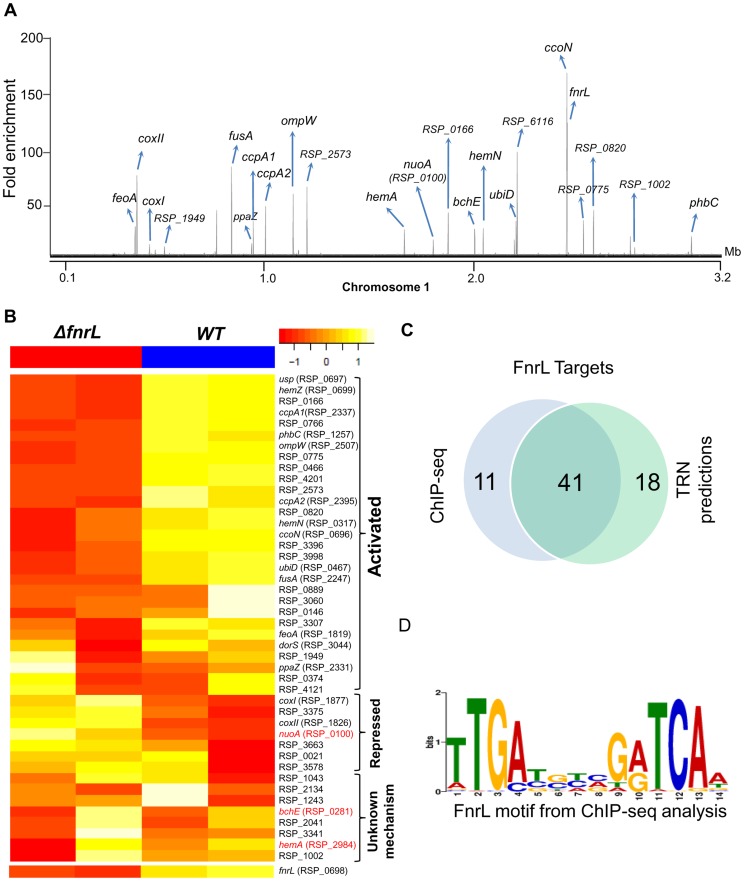
Analysis of the FnrL regulon in *R. sphaeroides*. (A) A total of 62 FnrL binding sites were identified by ChIP-seq across the *R. sphaeroides* genome. Binding sites across chromosome 1 are highlighted. MochiView [90] was used for visualization of binding profile. (B) Heat map depicts the differentially expressed FnrL target operons between wild type (WT) and *ΔfnrL* cells grown on acetate-based media. For brevity only the first members of the target operons are presented. The relative expression of *nuoA*, *bchE* and *hemA* (highlighted in red), which are known to be positively regulated by FnrL, are either not differentially expressed or differentially expressed in the opposite direction. (C) Venn diagram depicting the overlap between our FnrL ChIP-seq analysis and prediction from the large-scale reconstruction of *R. sphaeroides* transcriptional network. (D) Position weight matrix logo generated for FnrL using targets identified by ChIP-seq.

We determined the genome-wide FnrL binding sites using ChIP-seq with wild type (WT) cells grown under anoxygenic photosynthetic conditions. We reproducibly identified a total of 62 FnrL binding sites across 3 independent ChIP-seq experiments, corresponding to 52 known or predicted operons (S1 Table). These included several sites immediately upstream of genes involved in bacteriochlorophyll synthesis (*bchEJGP*), early steps in tetrapyrrole biosynthesis (*hemN*, *hemZ* and *hemA*), as well as genes that regulate anaerobic respiration using dimethyl sulfoxide (DMSO) as a terminal electron acceptor (*dorS*) (Fig. 1A, S1 Table). FnrL binding sites were also found upstream of genes encoding functions for iron transporter (*feoABC*) and iron sulfur cluster assembly (RSP_1949). When we compared this set of ChIP-seq identified FnrL binding sites to data from ChIP-chip analysis [24], 24 of the 27 FnrL binding sites identified previously were also detected in our analysis (S1 Table). The three previously identified FnrL binding sites not identified in our ChIP-seq analysis do not appear to contain a significant FnrL motif and likely represent false binding events. Furthermore, we found an additional 38 FnrL binding sites in the ChIP-seq dataset, implicating this TF as a direct regulator of a wide variety of new functions, ranging from protein synthesis and substrate transport to polyphosphate dependent phosphorylation and nitrogen metabolism (S1 Table, see Discussion). The larger number of sites identified in the ChIP-seq analysis compared to the previous ChIP-chip analysis for FnrL could be a reflection of the higher sensitivity and improved resolution obtainable with ChIP-seq analysis [30]. For instance, the higher signal to noise ratio of ChIP-seq could potentially allow for identification of relatively weak binding sites, which may be difficult to identify by ChIP-chip. Consistent with this, only 1 of the 24 ChIP-chip identified sites had lower than a 20-fold enrichment in the ChIP-seq dataset (S1 Table). On the other hand, some of these differences could also be the result of differences in peak calling algorithms and thresholds used to identify significant binding sites in the individual datasets.

In addition to the 49 FnrL binding sites that were identified upstream of operons, 13 binding sites were outside upstream regulatory regions of any annotated genes. These sites could represent non-functional sites or binding sites in the upstream regulatory regions of other unannotated genomic elements. For instance, 2 of these 13 additional sites were in putative promoter regions of recently identified sRNA in *R. sphaeroides* - RSs0019 and RSs2461 [31]. Thus, it is conceivable that these other unassigned binding sites are located in the upstream regulatory regions of other as of yet unidentified genomic elements. It is also worth noting that 41 of 59 (69.5%) FnrL target operons predicted in the large-scale TRN reconstruction [10] were verified via this ChIP-seq analysis (Fig. 1C), including 17 operons that were novel predictions in that TRN inference study (S1 Table).

To independently assess the functional role of FnrL in the regulation of target genes identified in our ChIP-seq analysis, we conducted microarray analysis to compare the gene expression of WT cells to a *ΔfnrL* deletion strain [9], [25] during growth on acetate as the sole carbon source, a condition we found that allows photosynthetic growth of both WT and *ΔfnrL* strains (S1 Figure). Consistent with FnrL being a global regulator, a total of 303 genes were differentially expressed between the 2 strains (cutoff – 1.5 fold change (FC), p<0.01), with 166 and 137 genes showing increased and decreased transcript abundance, respectively, between WT and *ΔfnrL* cells (S2 Table). Of the 48 operons in which we found FnrL binding via ChIP-seq (and for which probes exist on the *R. sphaeroides* gene chip), 24 were differentially expressed between WT and *ΔfnrL* cells (Fig. 1B), indicating that at least under the conditions assayed, the expression of these target genes is significantly influenced by FnrL. While the change in expression of some of the FnrL targets did not meet the significance cut-off used for this analysis (S2 Table), changes in their expression in response to the loss of FnrL was sufficient to allow a tentative assignment of their control by FnrL (S1 Table, Fig. 1B). The direct and potential indirect influence of FnrL on global gene expression are further detailed in the Discussion. It should also be noted that transcripts for *hemA* and *nuoA-N* (RSP_0100-12) did not show a significant difference in levels between WT and *ΔfnrL*, while transcripts encoding *bchEJGP* were elevated in *ΔfnrL* cells, despite the proposed positive role of FnrL in transcription of these operons [24]. These results suggest that transcription of some FnrL target operons might be under control of other TFs, wherein loss of FnrL is partially or fully compensated for by the activity of other transcriptional regulators in the *ΔfnrL* strain. Indeed, *hemA* transcription is known to also be directly activated by the response regulator, PrrA under anaerobic conditions [32], [33].

### PrrA—A global regulator of photosynthetic growth in *R. sphaeroides*

PrrA is the response regulator of the two component PrrAB system that has previously been proposed to be a major global TF in *R. sphaeroides* and related purple non-sulfur bacteria [20]. PrrA is essential for photosynthetic growth in *R. sphaeroides* and direct control of photosynthesis-related operons by PrrA has been shown via the use of *in vitro* experiments [22], [32]. To obtain a better understanding of the functional role of PrrA, we assessed PrrA activity using genome-wide gene expression data and ChIP-seq.

#### Redefining the set of PrrA target genes

Gene expression profiling experiments comparing mRNA abundance in a *ΔprrA* strain to WT cells were previously conducted under anaerobic respiratory conditions using DMSO as a terminal electron acceptor – a condition which permits growth of the photosynthesis deficient *ΔprrA* strain and under which PrrA is active in WT cells. This analysis showed that over 1000 genes were differentially expressed in the absence of PrrA [20]. However, a large percentage of these genes encoded functions related to protein synthesis and cell growth [20], suggesting that these might also reflect differences in growth rates between the 2 strains, possibly resulting from unlinked mutations in the *ΔprrA* strain. Consistent with this, we found that the *ΔprrA* strain used in the previous analysis (PrrA2) grew significantly faster than our WT strain under anaerobic respiratory conditions (S2A Figure). In contrast, an independently constructed markerless *ΔprrA* strain made for this study (PrrA3) grew similarly to WT under anaerobic respiratory conditions (S2A Figure) and showed similar pigmentation phenotypes to the original *ΔprrA* mutant. Consequently, we reassessed differences in gene expression between WT and *ΔprrA* using the PrrA3 mutant strain. We found a total of 255 genes were differentially expressed between WT and *ΔprrA* (2 FC, p<0.01) (S3 Table), significantly less than the 1058 previously reported at a similar cut off [20]. In addition, this set of 255 differentially expressed genes did not include any protein synthesis genes. We believe this set of differentially expressed genes, which are essentially a subset of those previously identified [20], provide a better picture of potential PrrA target genes.

Consistent with previous knowledge on PrrA, the 255 differentially expressed genes that we identified were enriched for genes known or predicted to be involved in photosynthetic processes (Table 1). In addition to photosynthesis related functions, other GO terms significantly enriched for differentially expressed genes in this dataset include categories such as the TCA cycle, electron transport chain and iron binding (Table 1). Overall, of the 255 differentially expressed genes (corresponding to 182 operons), mRNA levels from 148 were increased in the presence of PrrA, while 107 were decreased, supporting previous suggestions that PrrA functions as both a transcriptional activator and repressor [20].

**Table 1:** GO functional categories significantly enriched for genes regulated by PrrA.

GO ID	GO description	Number of genes in set	DE genes^a^	P-value^b^	Reg^c^
GO:0015979	Photosynthesis	34	20	0	+
GO:0022900	Electron transport chain	33	16	3.51E-13	+, −
GO:0006099	Tricarboxylic acid (TCA) cycle	11	6	6.01E-07	−
GO:0008299	Isoprenoid biosynthetic process	12	6	1.37E-06	+
GO:0018189	Pyrroloquinoline quinone biosynthetic process	4	3	1.17E-05	−
GO:0004129	Cytochrome-c oxidase activity	8	4	3.26E-05	−
GO:0033014	Tetrapyrrole biosynthetic process	5	3	5.56E-05	+
GO:0005506	Iron ion binding	52	10	1.62E-04	+, −
GO:0004497	Monooxygenase activity	17	4	2.32E-03	+, −

a The 255 differentially expressed genes obtained using a 2 fold-change cut off were utilized for this analysis.

b p-value based on the hypergeometric distribution

c Regulatory role PrrA on differentially expressed genes within the gene sets. +  =  positive regulation; −  =  negative regulation; +,−  =  some genes upregulated while others are downregulate in the gene set.

#### PrrA regulates transcription from only a subset of its binding sites

**Figure 2:**
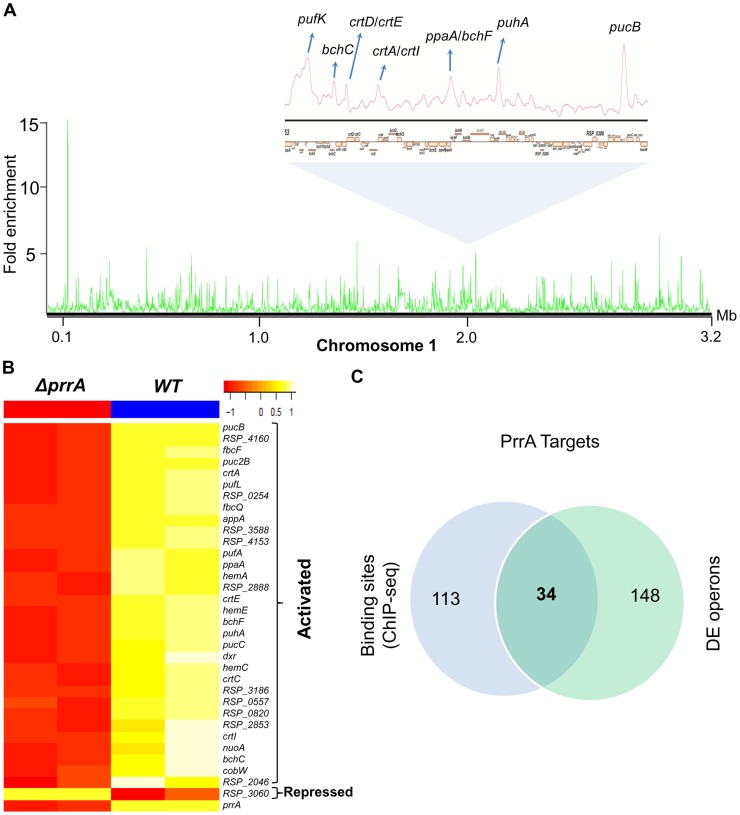
Analysis of the PrrA regulon in *R. sphaeroides*. (A) PrrA binding sites across chromosome 1. Binding sites within the photosynthetic gene cluster are enlarged. (B) Heat map depicting the PrrA targets genes from a pair-wise comparison of transcript levels from WT and *ΔprrA* cells grown under anaerobic respiratory conditions. (C) Venn diagram showing the overlap between identified ChIP-seq binding sites for PrrA and differentially expressed operons from microarray analysis. It should be noted that some binding sites are located between two divergently transcribed operons, which were both differentially expressed. In such cases, both operons were considered as direct PrrA targets.

To determine which differentially expressed genes are directly regulated by PrrA, we conducted ChIP-seq analysis on exponentially growing cells using a 3X myc-tagged PrrA protein that complements the photosynthetic growth defect of PrrA3 (S2B Figure). We observed significant enrichment for PrrA at ∼140 sites across the *R. sphaeroides* genome (Fig. 2A). Analysis of the sequences under all of these peaks did not reveal any strong consensus sequence shared by a significant number of these sites. Thus, to help determine the transcriptionally regulated direct targets of PrrA, only operons with both a significant peak and which were differentially expressed in PrrA3, were considered as candidate direct targets of this TF. A total of 34 operons met these criteria, including 18 photosynthesis related operons (Table 2, Figs. 2B and 2C). In addition to photosynthesis-related genes to which PrrA had previously been linked, these analyses indicate that PrrA is also a direct regulator of electron transport (regulating operons encoding *fbcFBC*, *fbcQ-soxDA* and RSP_0820 (cytochrome B561)), tetrapyrrole synthesis (*hemA*, *hemC* and *hemE*) and terpenoid backbone biosynthesis (*dxr*). Our data predict that all but one of these operons are positively regulated by PrrA, since RNA levels from these genes were lower in PrrA3 (Fig. 2B, Table 2). Other enriched sites in the genome not included in this set of transcriptionally regulated direct PrrA targets are provided in S4 Table.

**Table 2:** PrrA target genes identified by ChIP-seq and gene expression analysis of *R. sphaeroides* cells.

S/N†	GeneID	Gene Annotation*	chrID	Start^a^	Stop^a^	FC^b^	Reg^c^
1	RSP_0100-12	*nuoA-N* (NADH dehydrogenase)	chr1	1811600	1812599	2	+
2	RSP_0254	*DxsA*	chr1	1980200	1983799	2.9	+
	RSP_0257-5	*pufLMX* (Photosynthetic reaction center proteins)					+
	RSP_0258-RSP_6109	*pufKAB* (LHI alpha, Light-harvesting B875 protein)					+
3	RSP_0263-59	*bchCXYZ-pufQ* (Chlorophyll synthesis)	chr1	1988000	1988599	3	+
4	RSP_0265-4	*crtEF* (Carotenoid biosynthesis)	chr1	1990400	1990799	2.87	+
	RSP_0266-7	*crtCD* (Carotenoid biosynthesis)					+
5	RSP_0271-69	*crtIB-tspO* (Carotenoid biosynthesis)	chr1	1996400	1996799	2.57	+
	RSP_0272-5	*crtA-bchIDO* (Carotenoid biosynthesis)					+
6	RSP_0283	*ppaA* (Regulatory protein)	chr1	2010000	2010799	2.96	+
	RSP_0284-91	*bchFNBHLM-puhA* (Bacteriohlorophyll synthesis)					+
7	RSP_0290-1	*puhA* (Light-harvesting 1 (B870) complex assembly)	chr1	2019000	2019599	3.7	+
8	RSP_0314-RSP_6256	*pucBA* (LHII beta, light-harvesting B800/850 protein)	chr1	2042400	2043399	5.35	+
	RSP_0315	*pucC* (Light-harvesting 1 (B870) complex assembly)					+
9	RSP_0557	Hypothetical protein	chr1	2294400	2294599	2.2	+
10	RSP_0679	*hemC* (Hydroxymethylbilane synthase)	chr1	2424000	2424399	2.1	+
	RSP_0680	*hemE* (Uroporphyrinogen decarboxylase)					+
11	RSP_0820-RSP_6134	Cytochrome B561	chr1	2565800	2566199	2	+
12	RSP_1396-4	*fbcFBC* (Ubiquinol-cytochrome C reductase)	chr1	3174800	3175199	2.85	+
13	RSP_1518	*prrA* (Response regulator)	chr1	104400	105399	15.9	NA
14	RSP_1556-RSP_6158	*puc2B2A* (Light-harvesting complex)	chr1	146000	146799	3.4	+
15	RSP_1565	*appA* (Antirepressor of PpsR)	chr1	156200	156999	3.9	+
16	RSP_2046	Hypothetical protein	chr1	640600	642399	3.26	+
17	RSP_2687-90	*fbcQ-soxDA* (Cytochrome b-c1 subunit IV)	chr1	1332200	1332999	3.1	+
18	RSP_2709-10	*Dxr*	chr1	1357000	1357599	2.38	+
19	RSP_2829-24	*cobWNHIJK* (Cobalamin synthesis proteins)	chr1	1481600	1482199	2.7	+
20	RSP_2853-55	Transcriptional regulator	chr1	1451200	1451599	2.74	+
21	RSP_2888	*MppG*	chr1	1565000	1565199	1.7	+
22	RSP_2984	*hemA* (5-aminolevulinate synthase)	chr1	1674200	1676199	4.49	+
23	RSP_3060-3	Possible O-acetylserine synthase	chr2	101000	101399	2.27	−
24	RSP_3186	Hypothetical protein	chr2	239200	239999	3.49	+
25	RSP_3588-5	Hypothetical protein	chr2	680600	681599	4.74	+
26	RSP_4153-RSP_7385	Hypothetical protein	plasmidD	13600	14799	5	+
27	RSP_4160-2	Hypothetical protein	plasmidD	19600	20199	3.67	+

a Chromosomal locations of start and stop of ChIP-seq peaks.

b Fold enrichment of PrrA-myc ChIP over control myc antibody ChIP in WT control.

c Regulatory role of PrrA on target operons based on change in gene expression between WT and Δ*prrA* cells. +  =  positively regulated by PrrA. −  =  negatively regulated by PrrA. NA - Not applicable.

† Number of binding sites. Some binding sites correspond to more than one operon.

*dxsA - 1-deoxy-D-xylulose-5-phosphate synthase; *dxr* - 1-deoxy-D-xylulose 5-phosphate reductoisomerase.

### Analysis of newly predicted regulators of photosynthesis in *R. sphaeroides*

A recent large-scale reconstruction of the TRN of *R. sphaeroides* predicted that there are additional regulators of photosynthesis. Among the highest scoring TFs that fell into this category were: (i) CrpK (RSP_2572), a Crp/Fnr-family regulator, and (ii) MppG, a BadM/Rrf2-family protein. Using a combination of physiological, genetic and genomic analysis, we investigated the contributions made by these proteins to regulation of photosynthesis in *R. sphaeroides*.

### CrpK—A member of the Crp/FnrL family that controls many photosynthesis genes

CrpK is a Crp/Fnr-family TF, which based on Pfam analysis [34], shares similar cyclic nucleotide-binding and Crp-like helix-turn-helix domains as FnrL. However, unlike FnrL, CrpK does not possess the 4 cysteine residues at its N-terminus required for coordination with iron-sulfur clusters, suggesting CrpK might not directly sense oxygen. Nevertheless, ectopic expression of CrpK in an *ΔfnrL* mutant from an IPTG-inducible plasmid restores photosynthetic growth on succinate (S3A Figure), indicating CrpK might directly regulate a similar set of genes as FnrL. However, a markerless *crpK* deletion mutant is capable of photosynthetic growth on succinate (S3B Figure), indicating that FnrL and CrpK might also have distinct targets. In addition, CrpK transcript levels are ∼2 to 3 fold higher in photosynthetic cells relative to cells grown under aerobic or anaerobic respiratory conditions (indicating it may have a physiological role linked to photosynthesis), but the levels of CrpK-specific transcripts are lower than those coding for FnrL under all growth conditions that have so far been tested by global expression analysis.

#### The CrpK and FnrL regulons are overlapping but distinct

**Figure 3:**
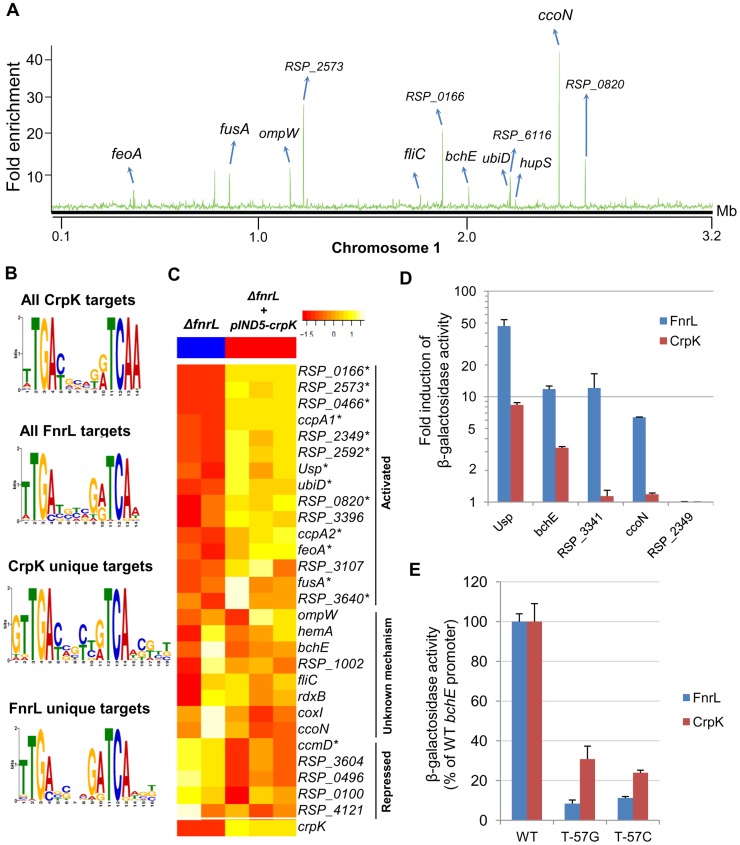
Analysis of the CrpK regulon in *R. sphaeroides*. (A) CrpK binding sites across chromosome 1. (B) Comparison of motifs generated for all CrpK and FnrL ChIP-seq identified targets, as well as those for targets exclusive to the CrpK and FnrL ChIP-seq datasets. (C) Pair-wise comparison of the global transcript level data between a *ΔfnrL* strain and a *ΔfnrL* strain over-expressing CrpK (*ΔfnrL*+pIND5-*crpk*) for all ChIP-seq identified CrpK target operons. Only the first members of the operons are shown. Significantly differentially expressed genes are indicated with *. (D) Fold increase in β-galactosidase activity observed after inducing either CrpK or FnrL synthesis using promoter-*lacZ* fusions of the *bchE*, RSP_0697 (Usp), *ccoN*, RSP_3341 and RSP_2349 promoters, integrated into the chromosome of a *ΔfnrL*-*ΔcrpK* reporter strain. Fold inductions represent fold change over the measured basal β-galactosidase activity prior to induction of CrpK or FnrL. (E) Percentage of β-galactosidase activity at mutant *bchE* promoters relative to the WT promoter. The average growth rate of cultures with FnrL induced was 3.4±0.2 hrs, while that of cultures with CrpK induced was 3.1±0.2 hrs. The error bars in (D) and (E) represent standard error of mean of 3 independent biological replicates.

We conducted ChIP-seq analysis using a 3X myc tagged variant of CrpK, which was able to restore photosynthetic growth to a *ΔfnrL* deletion strain (*ΔfnrL+*pIND5-*crpK*-3Xmyc) (S3A Figure) confirming its functionality (the observed difference in growth rate between the strains complemented with a tagged or untagged CrpK protein may reflect alterations in activity or abundance of the individual proteins). We identified a total of 38 binding sites for CrpK in the *R. sphaeroides* genome (Table 3, Fig. 3A). Consistent with its predicted involvement in regulation of photosynthetic genes, CrpK was found to bind to the upstream regulatory regions of *bchEJGP* and *hemA*, both known to be involved in the biosynthesis of photopigments or their tetrapyrrole precursors [35]–[37]. CrpK binding sites were also found upstream of genes encoding iron transporters (*feoABC, ccmD*) and iron-sulfur cluster binding proteins (*rdxBHIS*). In addition, 23 (60.5%) of the identified CrpK sites were also identified as FnrL target sites (Table 3, S1 Table, S4 Figure), possibly providing an explanation for the ability of CrpK to at least partially compensate for the loss of FnrL (S3A Figure). The remaining 15 CrpK binding sites were not occupied by FnrL under the conditions we tested (Table 3). On the other hand, FnrL was found bound to 39 sites that were not recognized by CrpK. These observations of TF occupancy for a subset of these overlapping and distinct sites were also verified via independent ChIP-qPCR analysis (S3D Figure), but it should be noted that many of the “CrpK unique sites” (i.e., those not also bound by FnrL) are relatively low enrichment sites in ChIP-seq assays (S4 Figure). However, this set of “CrpK unique sites” all possessed a similar shared motif to other identified CrpK sites, so we consider it likely that these are actually direct targets for control by this TF. The ChIP-seq peaks for CrpK and FnrL at sites bound by both TFs were centered at the same location for both TFs (S4 Figure) and consequently the predicted binding motifs for both TFs bear strong DNA sequence similarity at both shared and unique sites (Fig. 3B). This observation is consistent with general motif type recognized by Crp/Fnr-family TFs and the relatively high degree of amino acid sequence similarity in the predicted DNA binding motifs of CrpK and FnrL [24]. However, subtle differences between the motifs that are assembled by analysis of the “FnrL and CrpK unique sites” could be discerned, which might allow for future computational or experimental discrimination between target sites for each TF (Fig. 3B).

**Table 3:** CrpK binding sites across the *R. sphaeroides* genome identified by ChIP-seq.

S/N†	ID	Annotation	chrID	Start^a^	Stop^a^	FC^b^	Reg^c^
1	RSP_0069*	*fliC* (Flagellar filament protein)	chr1	1776800	1777199	3.84	
2	RSP_0100-12	*nuoA-N* (NADH dehydrogenase)	chr1	1812000	1812399	2.7	−
3	RSP_0166	TraR-like protein	chr1	1882200	1882599	22.6	+
4	RSP_0281-76	*bchEJGP* (Bacteriochlorophyll biosynthesis)	chr1	2007600	2007999	6.5	+
5	RSP_0466-4	Hypothetical protein	chr1	2201400	2201999	5.3	+
	RSP_0467-8	*UbiD*					+
6	RSP_0496*	*hupL* (Hydrogenase protein large subunit)	chr1	2232000	2232399	3.15	−
7	RSP_0692-89*	*rdxBHIS* (Iron-sulfur cluster-binding protein)	chr1	2436600	2436799	2.33	
8	RSP_0696-3	*ccoNOQP* (Cbb3-type cytochrome c oxidase)	chr1	2440200	2440799	41.4	+
	RSP_0697	Universal stress protein (Usp)					+
9	RSP_0820	Cytochrome B561	chr1	2565800	2566399	13	+
10	RSP_1002-3	Aspartate carbamoyltransferase	chr1	2760200	2760599	3.7	
11	RSP_1804*	*ccmD* (Heme exporter protein D)	chr1	388000	388399	3.42	−
12	RSP_1819-17	*feoABC* (Ferrous iron transport protein)	chr1	409200	409399	3.46	+
13	RSP_1877-6	Cytochrome c oxidase aa3 type (*coxI*)	chr1	477000	477399	3.61	−
14	RSP_2247	*fusA* (Elongation factor G)	chr1	862600	862999	9.77	+
15	RSP_2337	*ccpA1*	chr1	964200	964799	2.7	+
16	RSP_2349*	Hypothetical protein	chr1	975000	975399	3.2	
17	RSP_2395	*ccpA2* (BCCP, cytochrome c peroxidase)	chr1	1022400	1022799	3.13	+
18	RSP_2507	*ompW* (Outer membrane protein)	chr1	1152400	1152999	13.2	
19	RSP_2573	Hypothetical protein	chr1	1216800	1217999	21.26	+
20	RSP_2592*	Hypothetical protein	chr1	1234000	1234399	3.25	+
21	RSP_2984	*hemA* (5-aminolevulinate synthase)	chr1	1675800	1676199	2.83	
22	RSP_3107*	Hypothetical protein	chr2	152400	152999	3.27	+
23	RSP_3396-3	ABC opine/polyamine transporter	chr2	461000	461399	2.96	+
24	RSP_3604*	tRNA 2-selenouridine synthase	chr2	698600	698999	4.6	−
25	RSP_3640*	Hypothetical protein	chr2	748400	748599	3	+
26	RSP_4121	Hypothetical protein	plasmidC	88000	88399	2.94	−
27	RSP_6116	Hypothetical protein	chr1	2206600	2206999	8.7	NA
28	RSs0019	small RNA RSs0019	chr2	28600	28999	3.5	NA
29			chr1	403800	404199	5.26	NA
30			chr1	785400	785799	3.27	NA
31			chr1	792200	792799	10.18	NA
32			chr1	2193200	2193599	2.97	NA
33	*		chr1	2338000	2338399	3.1	NA
34	*		chr2	449800	450399	11.28	NA
35	*		chr2	856800	857399	7.1	NA
36	*		plasmidB	24000	24599	6.2	NA
			plasmidB				NA
37	*		plasmidD	17600	18199	5.56	NA
38	*		plasmidD	90000	90399	4.7	NA

*ChIP-seq binding sites bound by CrpK but not FnrL.

a Chromosomal locations of start and stop of ChIP-seq peaks.

b Fold enrichment of CrpK-myc ChIP over control myc antibody ChIP in WT control.

c Regulatory role of CrpK on target operons based on change in gene expression between WT and ΔcrpK cells. +  =  positively regulated by CrpK. −  =  negatively regulated by CrpK. NA - Not applicable (i.e., these genes are not represented on the *R. sphaeroides*)

† Number of binding sites. Some binding sites correspond to more than one operon.

#### CrpK controls expression of its predicted target genes

To independently test the role of CrpK on its predicted target genes, we conducted microarray analysis comparing the expression of a *ΔfnrL* strain to a *ΔfnrL* strain expressing an untagged *crpK* gene from an IPTG-inducible plasmid (*ΔfnrL+*pIND5-*crpK*) (S3A and S3C Figures), to bypass the effect of FnrL on these target operons (a *ΔfnrLΔcrpK* strain was not viable under any anaerobic or photosynthetic conditions that we tested). Of the 28 CrpK target operons identified by ChIP-seq (and for which probes exist on the *R. sphaeroides* gene chip), 14 of these were differentially expressed (using a 1.5 FC, p<0.01) under these conditions (Fig. 3C, Table 3), predicting that CrpK controls expression from these target promoters in this reporter strain. Transcripts from 13 of these differentially expressed operons were increased including those encoding RSP_0166 (a TraR-like protein), UbiD, RPS_0697 (universal stress protein, Usp) and iron transporter FeoABC, whereas 1 operon was down regulated in the absence of CrpK (Fig. 3C). Just as in the case of FnrL, regulatory control at some of the CrpK target operons such as *hemA, nuoA-N* and *bchEJGP* might be obscured by reprogramming of the transcriptional network in the *ΔfnrL* strain (see Discussion). Thus, the number of functional CrpK targets is likely larger. In general, the predicted direction of regulation by CrpK in all of the differentially expressed operons (activation or repression) were the same as observed with FnrL (Table 3, Fig. 3C).

Given the gene expression patterns observed above, we analyzed expression of promoter-*lacZ* fusions, using a few CrpK and FnrL target genes, in *ΔfnrLΔcrpK* double mutant reporter strains, as an additional test of the ability of these proteins to control activity of candidate promoters. Consistent with the predictions of our genome-wide analysis, we observed that CrpK was able to increase β-galactosidase activity from the *bchE*, universal stress protein (RSP_0697) and *ccoN* promoters, similar to FnrL though albeit with lower β-galactosidase activity (Fig. 3D). This result also indicates a positive regulatory role for both FnrL and CrpK have on *bchE*, a fact which was not possible to infer from our global gene expression data. No significant β-galactosidase activity was observed with the RSP_3341 promoter upon ectopic expression of CrpK (1.1 fold induction), whereas significant β-galactosidase activity was obtained when FnrL synthesis was induced. These data are also consistent with the ChIP-seq data, which indicates that FnrL, but not CrpK, binds to the RSP_3341 promoter (S4 Figure). For the one unique CrpK site tested in this analysis, RSP_2349, we did not observe any increase in β-galactosidase activity from this promoter when either CrpK or FnrL was ectopically expressed, suggesting that this CrpK binding site might not be functional under the growth conditions tested. Nevertheless, the ability of CrpK to bind the upstream regulatory regions of several FnrL target genes such as *bchEJGP* and *hemA*, as well as control their expression, provides a direct explanation for the ability of increased CrpK expression to restore photosynthetic growth to an FnrL mutant of *R. sphaeorides*.

#### CrpK and FnrL share similar DNA binding determinants

To test the above prediction that CrpK and FnrL can recognize related DNA sequences, we made 2 separate base substitutions in the predicted FnrL/CrpK consensus sequence of the *bchE* promoter, substituting thymine of the TCAA (at position −57 relative to the start codon) with either a guanine or cytosine. When these base substitutions were introduced into the *bchE* promoter fused to a promoterless *lacZ* gene, we found that they caused a significant decrease in β-galactosidase activity relative to the WT promoter when either FnrL or CrpK synthesis was induced (Fig. 3E). An ∼90% reduction in β-galactosidase activity was observed with both promoter mutations when FnrL synthesis was induced, while ∼70 and 80% decreases were observed with the individual guanine and cytosine mutations, respectively, when CrpK synthesis was induced. These data indicate that both TFs recognize similar sequences, consistent with predictions from the motif finding analysis and the fact that they belong to the same TF family.

### MppG—A newly identified modulator of photopigment biosynthesis

Another TF predicted by the large-scale TRN to play a role in the control of *R. sphaeroides* photosynthesis genes is the BadM/Rrf2 family TF, MppG. *mppG* transcript levels are increased under photosynthetic conditions in WT cells and *mppG* is predicted to be a direct target of PrrA (Table 2, Fig. 2B). Consistent with this, *mppG* transcript levels are more than 5 fold higher in WT cells relative to *ΔprrA*, being the most differentially expressed TF in that dataset (S3 Table). To test the role of MppG in regulation of photosynthesis, we conducted a combination of physiological, gene expression and protein-DNA binding assays for this TF.

#### MppG negatively regulates photopigment synthesis

**Figure 4:**
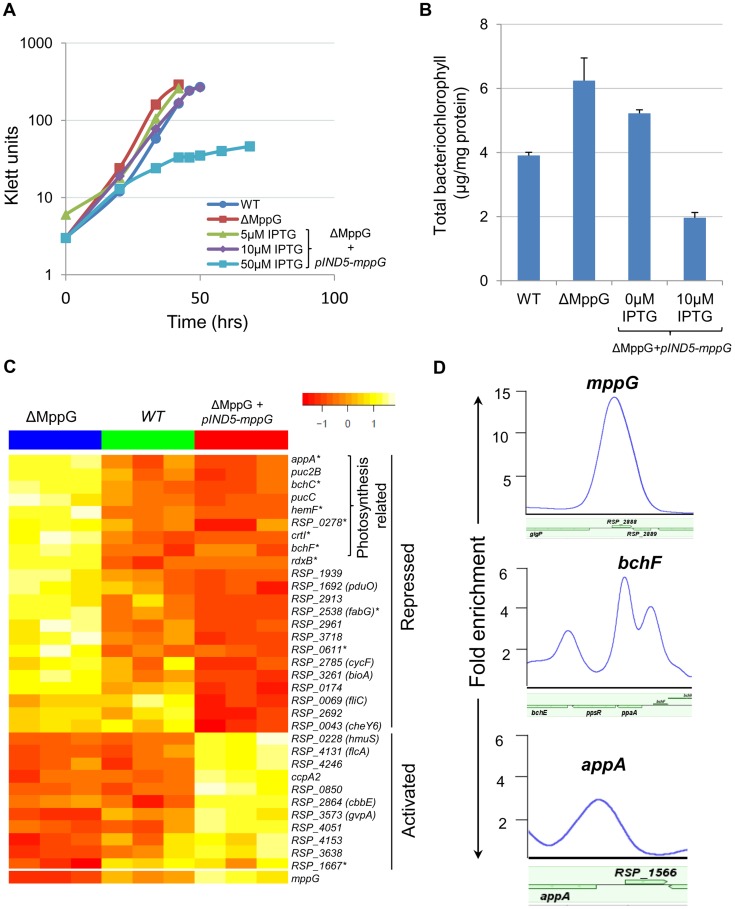
Physiological and genomic analysis of MppG regulation. (A) Growth of WT, ΔMppG and ΔMppG*+*pIND5-*mppG* with increasing IPTG concentrations under photosynthetic conditions. (B) Amounts of bacteriochlorophyll produced in WT, ΔMppG and ΔMppG*+*pIND5-*mppG*. (C) Expression profiles of genes differentially expressed in response to the loss of MppG (ΔMppG) or over-expression of MppG (ΔMppG*+*pIND5-*mppG*) strains. Genes differentially expressed in the ΔMppG only are indicated with an asterisk (*). (D) ChIP-seq binding profile of MppG at the *mppG*, *bchF* and *appA* promoters.

To assess the physiological role of MppG, we constructed and analyzed the properties of a *mppG* deletion mutant (ΔMppG). Furthermore, ΔMppG was complemented with *mppG* from an IPTG-inducible plasmid (ΔMppG*+*pIND5-*mppG*). The WT and ΔMppG strains both exhibited similar growth rates (Fig. 4A), while the complemented strains also grew at similar rates up to 10 µM IPTG, beyond which photosynthetic growth, but not aerobic growth, was severely negatively impacted (Fig. 4A, S5A Figure). This suggested a role for MppG in one or more aspects of photosynthesis.

It appeared that MppG had some impact on photopigment biosynthesis as there was a reduction of colony pigmentation when this protein was expressed from an IPTG-inducible plasmid. To test this hypothesis, we assessed the total amount of bacteriochlorophyll in cells containing or lacking MppG. These experiments showed that the ΔMppG mutant strain produces >50% more bacteriochlorophyll than its WT parent (Fig. 4B). Furthermore, ectopic expression of MppG lowered the amount of cellular bacteriochlorophyll (a 2-fold decrease from WT levels at 10 uM IPTG), providing additional support for the role of MppG as a regulator of photopigment synthesis (Fig. 4B). These data also indicate that MppG functions to negatively modulate photopigment synthesis.

#### MppG is a transcriptional repressor of photopigment and other photosynthetic genes

Given the observed negative role of MppG on photopigment synthesis, we conducted global gene expression analysis on the WT, ΔMppG and ΔMppG +pIND5-*mppG* strains under photosynthetic conditions. Consistent with the above observations, a variety of photosynthesis related genes were significantly differentially expressed between these strains. A total of 17 genes other than MppG were differentially expressed (cut-off −1.5 fold, p<0.05) between the WT and ΔMppG (Fig. 4C, S5 Table), with 12 of these genes having photosynthesis related functions. Most of these differentially expressed genes include genes from 7 operons (*appA*, *rdxBHIS*, *bchCXYZ*, *hemF*, *bchF*, *crtIB*, RSP_0278). Furthermore, transcript levels from all but one of the differentially expressed genes were down regulated in the presence of MppG, indicating that it functions as a transcriptional repressor. This predicted negative regulatory function for MppG is consistent with the decreased photopigment phenotype seen when this TF is over-expressed. Given that MppG transcript levels are increased under photosynthetic growth conditions relative to aerobic conditions in WT cells, its function is likely to fine-tune photopigment synthesis, similar to the predicted role for the sRNA, PcrZ [28].

By comparing the global gene expression profile of the ΔMppG strain with that of cells ectopically expressing this TF, we found a total of 36 genes differentially expressed (1.5 FC, p<0.05), including 14 of the 17 genes that were differentially expressed between WT and ΔMppG (S6 Table). The other 22 genes that were differentially expressed when MppG was ectopically expressed included additional photosynthesis related genes like those encoding light-harvesting proteins (*pucC* and *puc2B*), as well as genes involved in functions ranging from iron and heme transport to fatty acid biosynthesis (Fig. 4C, S6 Table). Twenty six of these 36 differentially expressed genes were also down regulated when MppG was ectopically expressed, consistent with this TF functioning as a repressor of photosynthesis and other functions.

#### MppG directly represses transcription of genes encoding photosynthesis related proteins

To further test the predicted direct role of MppG in regulation of photosynthesis, we performed 2 independent ChIP-seq analyses using a ΔMppG strain containing a 3X myc-tagged variant of MppG that complements the phenotype of the parent strain (ΔMppG*+*pIND5-*mppG*-3Xmyc) (S5B Figure). This ChIP-seq analysis identified a total of 52 MppG binding sites across the genome. We found that most of the genes downstream of these binding sites were not differentially expressed in the presence or absence of MppG in any of global gene expression datasets. In addition, we were unable to identify any conserved DNA motif shared by a significant number of these target sites. Thus, to identify potential direct targets of MppG, only operons that were both differentially expressed in either of our global gene expression datasets and had a significant ChIP-seq peak were considered. When these criteria were applied, we identified 9 potential MppG target operons: *bchCXYZ*, *bchFNBH*, *rdxBHIS*, *appA*, RSP_1257-4, RSP_2692, *mppG*, RSP_2961 and RSP_3718 (Table 4, Fig. 4D). Transcript levels from all of these operons were lower in the presence of MppG and they represent the most high confidence direct MppG targets in our dataset. The 43 other sites which showed significant enrichment for MppG are provided in S7 Table.

**Table 4:** MppG target genes identified by ChIP-seq and gene expression analysis.

	ID	Annotation	chrID	Start^a^	Stop^a^	FC^b^	Reg^c^
1	RSP_0263-59	*bchCXYZ-pufQ*	chr1	1988000	1988199	2	−
2	RSP_0284-91	*bchFNBH-chlL-bchM-puhA*	chr1	2010200	2010799	3.23	−
3	RSP_0692-89	*RdxBHIS*	chr1	2436600	2436999	3.35	−
4	RSP_1257-4	Polyhydroxyalkanoic synthase	chr1	3026800	3027199	4.3	−
5	RSP_1565	*AppA*	chr1	156400	156599	2.75	−
6	RSP_2692	Acyltransferase domain (LPS)	chr1	1338400	1338799	2.61	−
7	RSP_2888	*mppG*	chr1	1564600	1565599	17.4	NA
8	RSP_2961	Protein containing a CBS domain	chr1	1643600	1643799	2.45	−
9	RSP_3718	Hypothetical protein	chr2	841800	842199	2.24	−

a Chromosomal locations of start and stop of ChIP-seq peaks.

b Fold enrichment of MppG-myc ChIP over control myc antibody ChIP in WT control.

c Regulatory role of MppG on target operons based on change in gene expression between ΔMppG and WT or ΔMppG*+*pIND5-*mppG* cells. −  =  negatively regulated by MppG. NA - Not applicable.

## Discussion

**Figure 5:**
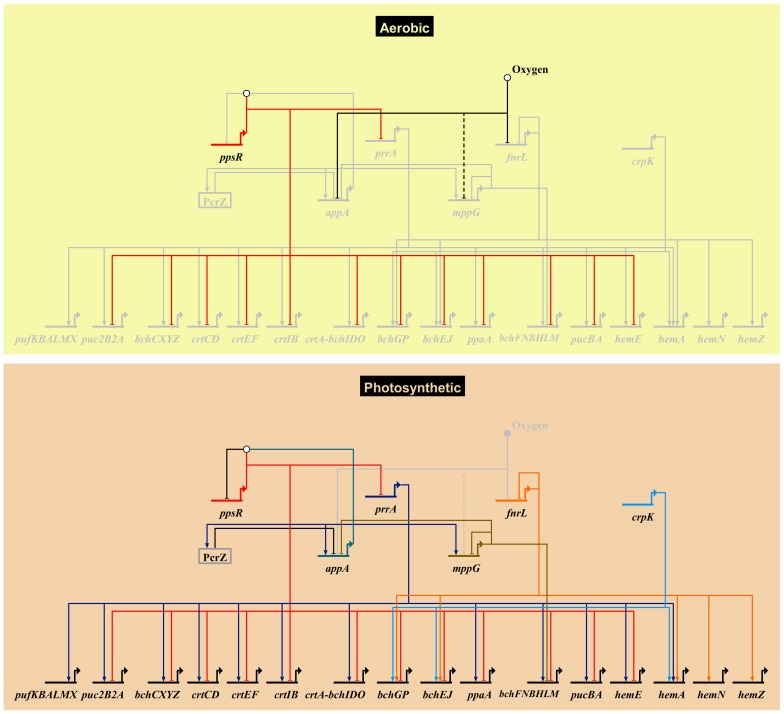
Photosynthetic gene regulatory network. An overview of the *R. sphaeroides* photosynthetic gene regulatory network, showing known transcriptional regulators and their photosynthesis-related direct target genes. Grey nodes and edges indicate inactive genes and interactions. The top panel (Aerobic) depicts the regulatory control mechanism of photosynthesis genes under aerobic respiratory conditions in the dark. Under this condition the anti-repressor protein AppA, which has been proposed to be sensitive to oxygen and light, is inactivated. This allows its cognate repressor, PpsR, to downregulate the expression of several photosynthesis related genes including the gene encoding the transcription factor PrrA. This results in the inhibition of photosynthesis by preventing the production of photopigments. Molecular oxygen also inhibits the activity of the transcription factors FnrL and potentially MppG (the uncertain nature of effect is indicated by a dashed edge). Under anoxygenic photosynthetic conditions (bottom panel), AppA becomes active and directly interacts with PpsR(depicted by the white circle above *ppsR*) inhibiting its activity. In addition, the activators of photosynthesis, PrrA, FnrL and CrpK, become active under these conditions and induce the expression of photosynthetic genes. Under these conditions, the photopigment gene repressors MppG and the sRNA PcrZ are also active, negatively modulating photopigment gene expression. The expression of *appA* and the activity of its gene product is dependent on regulatory inputs from PrrA, MppG, PcrZ and oxygen. Biotapestry was used for network visualization [91].

Our analyses have provided new information on the TRN controlling bacterial photosynthesis in *R. sphaeroides*. We confirmed the predicted involvement of two previously uncharacterized TFs, CrpK and MppG, in the regulation of photosynthesis related genes. We also extended the regulons of PrrA and FnrL, which had previously been implicated in regulation of the photosynthetic lifestyle of *R. sphaeroides*. Our analyses, combined with previous analyses of PrrA, FnrL and PpsR, illustrate the depth, complexity and robustness of the photosynthetic TRN. They also highlight significant combinatorial regulation of target genes, cross-talk between regulators and redundancy in the use of TFs within this network (Fig. 5), features that are likely to be broadly seen in the TRN of other biological systems.

### New insights into regulation by PrrA and FnrL

Previous analysis of photosynthetic gene expression in *R. sphaeroides* had established the importance of 3 global TFs, PpsR, PrrA and FnrL, in the regulation of this lifestyle [8], [9], [19], [20], [24], [26], [27]. Our global analysis of these previously identified members of this TRN extends prior knowledge by comprehensively identifying the direct targets for two of these proteins, PrrA and FnrL. The work in this paper also, complements parallel genome-wide analysis of a global repressor of photosynthesis, PpsR [10].

For example, our analysis indicated the total number of genes, directly or indirectly, controlled by *R. sphaeroides* PrrA is ∼4 times smaller than previously reported, providing a picture of the PrrA regulon that is not influenced by apparent growth-rate differences between wild type and the mutant used previously [20]. In addition, our data verified the major direct role played by this TF in photosynthesis gene expression, significantly expanding previous analyses that reported the ability of PrrA to bind DNA *in vitro* at a handful of sites [32], [38], [39] Our data show that PrrA, controls expression of genes required for light energy capture, as well as a number of operons encoding proteins involved in electron transport both directly (e.g., *fbcFBC* complex, cytochrome B561 and *cycA* [40]) and indirectly (S3 Table).

Although we identified several new PrrA direct targets, we were unable to identify a strong consensus binding motif for this TF. While PrrA, and its analog in *Rhodobacter capsulatus* RegA, have been proposed to bind a degenerate GCG inverted repeat with a varying length spacer region, previous analyses have suggested that both DNA curvature and sequence specificity might contribute to target site recognition [23], [32]. These potential features, together with the GC-rich nature of the *R. sphaeroides* genome and the Fis-like nature of the PrrA DNA binding domain [23], possibly made it difficult to identify a shared motif among target genes from our analysis. Thus, despite identifying members of the PrrA regulon, it is not always possible to predict the consensus binding site for a given TF.

A large-scale reconstruction of the *R. sphaeroides* TRN predicted that the regulon of a second mater regulator, FnrL, was larger than previously described [24]. Our studies verified several of these predictions, significantly extending the size and function of genes in the FnrL regulon to include nitrogen regulatory proteins, iron sulfur assembly proteins, ABC transporters, additional TFs and recently identified sRNAs, all of which significantly increase the scope of genes and functions that are controlled by FnrL (see below). This illustrates how the predictions of a large-scale TRN can provide new insights into the functions that are regulated by even a previously well-studied TF.

### Redundancy of the photosynthetic TRN

One of the previously uncharacterized TFs we tested for a role in the photosynthetic lifestyle was CrpK. Genome-wide analysis of CrpK targets revealed an overlapping but distinct regulon to that of FnrL, providing an explanation for the ability of CrpK to rescue the photosynthesis defect of an FnrL deletion strain.

While bacterial TRNs are often tightly controlled, they also need to be robust to allow cells to adapt to potentially deleterious changes to these networks. Given the central role of FnrL in regulating photosynthesis and a large number of anaerobic processes, the redundancy observed between this TF and the CrpK regulon might function to provide robustness to the *R. sphaeroides* photosynthetic TRN. Alternatively, CrpK might have a broader function under different conditions from FnrL. For instance, FnrL contains an O_2_-sensitive iron sulfur cluster that controls it DNA binding activity [24], so the absence of such a metal center in CrpK might allow this protein to function under conditions that would inactivate FnrL, facilitating photosynthesis or other metabolic functions during microaerophilic or semi-aerobic growth in nature. Interestingly, while FnrL binds upstream of *dorS* (encoding the histidine kinase of the DorSR two-component system involved in regulation of anaerobic respiratory growth on DMSO [41]), CrpK binding was not observed at this promoter (S4 Figure). This suggests that CrpK's ability to functionally replace FnrL might not extend to FnrL's role in regulation of anaerobic respiration.

In order to assess predicted cases of redundancy in a TRN or overlapping target genes of TFs, it is important to identify promoter elements that may allow discrimination between binding sites. For instance, although the predicted consensus motifs derived from the FnrL and CrpK binding sites were similar, the observation that both proteins can recognize unique, as well as overlapping sites, indicates there must be some subtle but functionally significant differences in DNA recognition by these TFs. Closer inspection of these DNA sequence motifs, suggest there may be a greater tolerance by FnrL for deviations from the TGA N_6_ TCA consensus, while bases within the spacer region or outside the core target site might play a role. Under the anaerobic photosynthetic growth conditions typically used in the lab, the CrpK transcript is present at a significantly lower level than that of FnrL. While it is possible that both proteins might compete for some shared binding sites under laboratory conditions, our analysis of a few shared or unique promoters suggested this was likely not a major factor in our experiments (S3E Figure). However, reproducibly greater FnrL enrichment was observed at the *ccoN*/RSP_0697 promoter in the absence of CrpK (S3D Figure), so the possibility that multiple TFs can compete for binding at selected sites cannot be ruled out without additional genetic and biochemical studies. This illustrates the need to couple predictions of TRN function with experimental studies.

### Incoherent feed forward regulation of the photosynthesis TRN

The second photosynthesis-related TF characterized for the first time in this study was MppG. Our data showed that MppG functions as a direct transcriptional repressor of photopigment biosynthesis operons, including *bchCXYZ* and *bchFNBHM*, with high cellular levels of this protein inhibiting photosynthetic growth. In addition, transcripts from several other operons that encode photosynthesis-related functions were indirectly repressed by MppG. Our data predict that much of this indirect regulation of photosynthesis function is achieved through the direct regulation of the gene that encodes the anti-repressor, AppA, by MppG. Reduced cellular levels of AppA caused by the presence of MppG, would in principle cause accumulation of free PpsR under photosynthetic conditions, which would lead to repression of the photosynthesis-related genes that are PpsR targets (Fig. 5). Given that *mppG* transcript levels are significantly elevated during photosynthetic growth, its function in repressing photopigment synthesis would appear to be counterintuitive, similar to the observation for the sRNA, PcrZ [28]. Since no significant difference in photosynthetic growth was observed between WT and ΔMppG cells, the additional pigment produced in the ΔMppG mutant strain did provide increased fitness, potentially equating to a waste of cellular resources in the production of this extra pigment. In addition, the presence of excess photopigment could be a source of metabolic stress, especially since they can result in production of reactive oxygen species if light is present under microaerophilic conditions in the lab or in nature [42]. Thus, MppG may function as a negative modulator of pigment synthesis to ensure the optimal expression and tight coordination between expression of photopigment biosynthetic pathway genes and those for other components of the photosynthetic apparatus.

### Newly-identified links between photosynthetic and iron homeostasis gene regulatory networks

In addition to its role in photosynthesis, MppG also regulates, either directly or indirectly, a variety genes encoding iron/heme dependent proteins (AppA, RdxBHIS, BchX, BchL, RSP_2785) and iron/heme transporters (RSP_2913, HmuS). MppG shares a high degree of amino acid sequence similarity to RirA, which was previously shown to regulate iron-responsive genes in *Rhizobium leguminosarum* [43], [44]. Thus, in addition to its role in regulation of photopigment synthesis, MppG appears to have a previously unidentified role in maintaining iron homeostasis during photosynthetic growth in *R. sphaeroides*. Furthermore, like RirA, MppG possesses a set of cysteine residues in its C-terminal region, which could coordinate an iron-sulfur cluster or some other metal, potentially allowing it to directly sense signals such as oxygen or metal availability.

Our data also provide new evidence that both FnrL and CrpK directly regulate genes encoding iron-dependent, iron transport and iron-sulfur biogenesis proteins, as well as several proteins involved in tetrapyrrole biosynthesis. In addition, we showed that FnrL directly activates expression of another RirA-like protein, RSP_3341, which has also been shown to directly regulate other iron dependent genes in *R. sphaeroides* [10]. Thus, we have provided new evidence that the TRNs and TFs controlling photosynthesis and iron homeostasis are tightly linked in *R. sphaeroides*. This link is likely, at least in part, due to the anaerobic anoxygenic mode of photosynthesis in *R. sphaeroides*, the sensitivity of Fe-S clusters to oxygen, and the involvement of a variety of iron-dependent proteins in light energy capture or other aspects of photosynthesis.

### Cross-talk between TFs that regulate photosynthetic gene expression

For complex TRNs to function effectively, the components of the network often need to communicate with one another, and this is the case with the photosynthetic TRN. For example, our previous analysis of the PpsR regulon identified a PpsR binding site upstream of *prrA*, in an intra-operonic promoter shown to be occupied by σ^70^ under photosynthetic conditions [24]. If this PpsR binding site upstream of *prrA* is functional, it would provide an additional, previously unrecognized, mechanism to prevent aerobic expression of photosynthetic genes. We also found that PrrA directly activates both AppA and MppG, which in turn represses AppA, forming an incoherent feed-forward loop to control photosynthetic genes (Fig. 5) similar to the situation proposed for PcrZ [28]. Thus, our data provide new support for the previous hypotheses that the control of *appA* transcription serves as a major point of integration of regulatory signals, integrating opposing regulatory inputs from PrrA, MppG, PcrZ, oxygen and possibly other as of yet unidentified factors. Type 1 incoherent feedforward loops can enable significant acceleration in the expression of a target gene in response to a signal compared to simple activation [45]. Thus we predict that this network architecture likely results in a rapid response of cells to small environmental perturbations and allows optimal expression of photosynthetic genes under anaerobic conditions.

### *R. sphaeroides* photosynthetic TRN as a multi-faceted network

**Figure 6:**
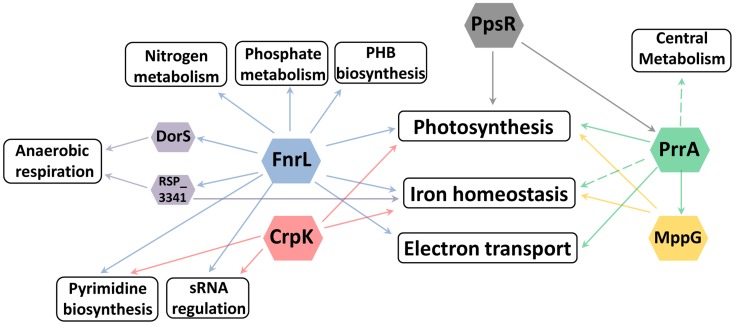
Cellular processes regulated by photosynthesis global regulators. Overview of the different cellular processes coordinated by the TFs regulating photosynthesis in *R. sphaeroides*.

Bacteria and other cells use a myriad of TRNs to respond to different types of stimuli, with these TRNs varying in depth and complexity [46], [47]. While the regulation of some cellular processes can be largely controlled by a TRN involving just one TF (for instance, LexA regulation of DNA repair in some bacteria [48]), other cellular processes involve the coordinated activities of multiple globally and locally acting TFs (e.g., the regulation of amino acid metabolism by ArgR, Lrp and TrpR in *E. coli* [49]). Generally, cellular processes which result in significant physiological or morphological changes (such as sporulation in Bacillus [50]–[53]) or are central to cell survival (such as central metabolism [54] or chemotaxis [55]) require highly interconnected TRNs involving multiple TFs and sensory components. The TRN network controlling photosynthesis in *R. sphaeroides* is multi-faceted, involving the activities of at least 5 TFs (including 4 global regulators) and one sRNA (Figs. 5, 6).

It is not surprising to find that a photosynthetic TRN has these multiple elements given the number and types of functions that need to be coordinated to harvest and conserve the energy in sunlight. In addition, the transition to the photosynthetic lifestyle of *R. sphaeroides* involves profound changes gene expression [56], resulting in major physiological changes that culminate in the accumulation of photopigments and formation of specialized intracytoplasmic membrane structures to house the photosynthetic apparatus [57]. Achieving such seamless transitions requires the use of a multi-faceted TRN, parts of which have been described in this work.

Some aspects of the TRN controlling photosynthesis in *R. sphaeroides* show similarities to mechanisms employed in other photosynthetic cells, suggesting that design principles learned from this system will be applicable to other organisms. For instance, in the cyanobacterium *Synechocystis* sp., regulation of photosynthetic gene expression has been shown to be under the control of bacterial two-component systems, which are proposed to sense the redox state of the cell and coordinate the expression of the two photosystems (PSI and PSII) to maintain redox poise [58]–[60](a process referred to as photosystem stoichiometry adjustment [61]). In this organism, loss of the response regulator RppA or its cognate sensor kinase RppB, has been shown to result in dysfunctional regulation of PSI and PSII in response to changes in the redox state of the plastoquinone pool [58]. In addition, the sensor kinase Hik2 has also been proposed to sense redox signals [60] and activate its cognate response regulator Rre1 in cyanobacteria [62]. Homologs of Rre1 have been shown to bind to and regulate the expression of photosynthesis related genes in other organisms [60]. Hik2 has also been shown to interact with RppA, potentially controlling its activities in regulation of photosystem stoichiometry [62]. These characteristics could make these systems functionally analogous to the PrrAB system in *R. sphaeroides*, which has also been proposed to regulate photosynthetic gene expression in response to redox state of the ubiquinone pool [63]. A third cyanobacterial sensor kinase Hik33 is proposed to function as global regulator integrating multiple inputs from different environmental stress conditions including cold and nutrient stress, and high light intensities [60], [64]. Its cognate response regulator Rre26 has been shown to bind and/or regulate the expression of specific photosynthesis related genes [65], [66]. Thus, through the use of two-component systems sensing the cell's redox state, cyanobacteria are able to control expression of their photosynthetic apparatus.

Cyanobacteria are the ancestors of chloroplasts found in modern day algae and higher plants [61]. While the TRNs controlling photosynthetic gene expression are not as well characterized in plants, the nuclear-encoded homolog of Hik2, CSK (a chloroplast sensor kinase which has lost the conserved histidine residue that serves as the site of phosphorylation in bacterial sensor kinases), has been shown to be required for the normal expression of PSI and PSII in response to changes in the redox state of the chloroplast plastoquinone pool in *Arabidopsis thaliana* [61], [67]. While no cognate response regulator has been identified for CSK, it has been shown to directly interact with the chloroplast-encoded sigma factor SIG1 and PTK (plastid transcription kinase). CSK is proposed to phosphorylate SIG1 when the plastoquinone pool is oxidized resulting in transcriptional repression of PSI genes, while permitting expression of PSII genes [67]. Thus, aspects of the regulatory mechanisms that control transcription of photosynthetic genes appear to be conserved between purple bacteria, cyanobacteria and photosynthetic eukaryotes.

One link between cellular redox state and gene expression is achieved through the two-component PrrAB system in *R. sphaeroides*. However, in addition to this TF, our studies show that *R. sphaeroides* employs at least 4 other TFs to control photosynthesis. The requirement for these additional TFs is likely, in part, due to the anaerobic nature of photosynthesis in *R. sphaeroides*, requiring its photosynthetic TRN to incorporate additional systems that sense oxygen tensions and modulate gene expression to minimize production of reactive oxygen species that can damage cellular components [42]. Thus, the *R. sphaeroides* photosynthetic TRN likely integrates the signals of cellular redox state, oxygen tension and possibly light intensity [68] to achieve optimal expression of photosynthesis genes. It should be noted that in general, the TRNs controlling photosynthesis in cyanobacteria and higher plants, are not as well characterized as that of *R. sphaeroides* and thus the complexity of these systems are probably yet to be fully appreciated.

### Coordinated regulation of other pathways by global transcriptional regulators of photosynthesis

Historically, TFs were often identified while studying individual metabolic or developmental pathways. Our results illustrate how the ability to globally predict and analyze the roles of TFs provides insight into previously unknown roles and connections between these proteins. For example, in characterizing the regulons of global regulators involved in the transcriptional control of photosynthesis in *R. sphaeroides*: PrrA, FnrL and CrpK, we found that each of these TFs directly or indirectly regulates a broad range of other cellular processes. Of the total number of operons directly regulated by these TFs, only 18 (∼53%), 5 (∼6%) and 3 (∼10%) (for PrrA, FnrL, and CrpK respectively), correspond to operons directly involved in photosynthesis, suggesting that these TFs may coordinate the control of photosynthesis with other processes that are beneficial or even required for the photosynthetic lifestyle. In addition to the previously noted example of iron homeostasis, FnrL is predicted to directly regulate the expression of the nitrogen regulatory proteins GlnB and GlnK (homologs of nitrogen regulatory protein P-II), which modulate the synthesis and activity of glutamine synthetase, implicating FnrL in the regulation of nitrogen metabolism [69], [70]. In addition, both FnrL and CrpK are predicted to directly regulate the expression of aspartate carbamoyltransferase (RSP_1002), which catalyzes the first step in the pyrimidine biosynthetic pathway. Furthermore, FnrL and CrpK also both directly regulate key enzymes in the electron transport chain including NuoA-N (RSP_0100-12) and CcoNOQP (Cbb3-type cytochrome c oxidase) (Table 3, S1 Table). On the other hand, PrrA is predicted to directly control the expression genes encoding a variety of electron transport chain enzymes including *nuoA-N* (RSP_0100-12), *fbcFBC*, *fbcQ* and cytochrome B561 (Table 2).

In addition to these metabolic functions, PrrA and FnrL are also predicted to directly regulate the expression of a number of other transcriptional regulators. For instance, PrrA directly activates both MppG and RSP_2854 (a TetR family TF) in addition to the anti-repressor of PpsR, AppA. On the other hand, FnrL is predicted to directly regulate 4 other TFs: DorS (a direct regulator of DMSO reductase), RSP_3341 (a direct regulator of nitrate reductase), RSP_4201 (an ArsR family TF) and RSP_1243 (a LacI family TF), as well as 2 proposed small RNAs (RSs0019 and RSs2461). While the cellular roles of some of these putative transcriptional regulators are unknown, the expression of their target genes is likely to be indirectly affected by FnrL and PrrA under appropriate conditions. Consistent with this, over 85% of the 303 differentially expressed genes between WT and ΔFnrL cells did not correspond to direct targets of FnrL. Instead this set of indirectly regulated genes included known direct target of DorS and RSP_3341, a variety iron transport and iron-dependent genes, genes involved in electron transport (such as quinol oxidases), nitrogen metabolism, and several photosynthetic genes. The set of indirectly controlled PrrA genes included those encoding several enzymes involved in central carbon metabolism (TCA cycle) and electron transport. These data predict that, through direct and indirect mechanisms, FnrL and PrrA serve to integrate and coordinate of the processes of photosynthesis, central metabolism, nitrogen metabolism, anaerobic respiration, electron transport, pyrimidine biosynthesis, PHB biosynthesis, phosphate metabolism and iron homeostasis during anaerobiosis (Fig. 6). These findings provide insight into previously unrecognized processes controlled by these TFs that could potentially be conserved by homologues of these TFs in other organisms.

In *E. co*li, the sRNA FnrS is directly regulated by FNR [71] and functions to repress several target RNAs under anaerobic conditions [72]. The two sRNAs (RSs0019 and RSs2461), which we found to be direct targets of FnrL, are yet to be functionally characterized [31] and do not share sequence similarities to FnrS. Additionally, the regulatory influence of FnrL on these target sRNAs could not be established in this study, as they are not represented on the *R. sphaeroides* Affymetrix gene chip used in our analyses. Nevertheless, one might also expect that the indirect targets of FnrL captured in our global expression analysis also includes downstream targets of these regulatory elements.

Interestingly, only about 50% of the genes predicted to be directly regulated by FnrL and CrpK were observed to be differentially expressed when the expression profile of the cognate deletion mutants were compared to that of wild type cells. Similar observations have previously been reported for another Crp family protein, FNR, in *E. coli* [71]. Thus, these observations could reflect the requirement of as yet uncharacterized TFs, which could function as condition-dependent co-activators, for controlling the expression of their target genes [71]. Alternatively, these observation could be the result of condition-dependent repression of specific operons by alternative TFs that obscured the regulatory influence of FnrL and CrpK on their target promoters under the specific conditions used for our global gene expression assays. Our analyses have also shown that several of the photosynthetic operons are under the control of multiple TFs, raising the possibility that the regulatory effect of each of these TFs, could potentially be compensated for by others at some of these operons, as shown for FnrL and CrpK. If this is the case, it could equip the cells with increased robustness in the expression of specific operons, obscuring the regulatory influence of individual TFs. This feature of the TRN would better enable cells to adapt to potentially deleterious changes.

Several of the genomic locations that were enriched for PrrA and MppG binding also did not show significant changes in gene expression under the conditions we tested and thus were not considered further as direct targets of these TFs (see Results). In addition, we observed that the TF enrichment at target sites was much lower for PrrA and MppG, than at FnrL and CrpK sites. These observations could reflect fundamental differences in the binding properties of these TFs. For instance, the predicted NMR structure of the PrrA DNA binding domain indicates that it is a Fis-like protein [23]. Previous studies in *E. coli* have shown that Fis, a nucleoid associated protein, binds to DNA in both a sequence specific and non-specific manner [73], [74] and that only about a fifth of bound sites are differentially expressed upon deletion of Fis [73]. If PrrA exhibits similar properties, it could account for the large number of bindings sites observed in our ChIP-seq data that do not correspond to genes that are differentially expressed in a PrrA-dependent manner.

### Concluding remarks

Using a combination of genetic, genomic and physiological approaches, guided in large part by computational predictions from a large-scale reconstruction of the *R. sphaeroides* TRN, we obtained a significant amount of new knowledge about regulation of photosynthesis in *R. sphaeroides*. Our analyses highlight the important role computational predictions can play in guiding biological discovery, as novel components of the photosynthetic TRN, not previously identified using traditional approaches, were identified computationally, with those predictions serving as the basis for this work. We expect that predictions from this large-scale TRN will continue to provide new insights into other aspects of *R. sphaeroides* diverse metabolic and energetic lifestyles, including those involved in production of high-value commodities such as biofuel precursors. In addition, given the ancestral relationship of *R. sphaeroides* to plants and other oxygenic phototrophs, we predict that knowledge of this photosynthetic TRN will help inform parallel or future studies in other photosynthetic organisms. Integration of the available large-scale network models of metabolism and transcriptional regulation for *R. sphaeroides*, will broaden the predictive capabilities of these models and further guide future experimental efforts.

## Materials and Methods

### Bacterial strains and growth conditions

*R. sphaeroides* 2.4.1 was used as the parental (wild type) strain and all mutants were constructed in this background except the *ΔfnrLΔcrpK* double deletion strain which was constructed in an existing *ΔfnrL* mutant background [9] (S8 Table). *E. coli* DH5α was used as a plasmid host, and *E. coli* S17-1 was used to conjugate DNA into *R. sphaeroides*. *R. sphaeroides* cultures were incubated at 30°C in Sistrom's minimal medium (SMM) [75]. Anaerobic cultures were started from cells obtained from exponentially growing aerobic cultures. When required, the media was supplemented with 100 µM IPTG, 25 µg/mL kanamycin, 25 µg/mL spectinomycin, or 1 µg/mL tetracycline. *E. coli* cells were grown in Luria Bertani medium at 37°C, supplemented with 50 µg/mL kanamycin, 25 µg/mL spectinomycin, or 20 µg/mL tetracycline where needed [11].

### Quantification of bacteriochlorophyll

Photosynthetic pigments were quantified in *R. sphaeroides* strains grown photosynthetically in screw cap tubes at a light intensity of ∼10 W/m^2^ as previously described [76]. Briefly, 5 mL of culture was centrifuged and supernatant discarded. Cells were resuspended in 100 µL of water, transferred to 15 mL glass corex centrifuge tubes held in centrifuge adaptors and covered with rubber stoppers to prevent exposure to light. 4.9 mL of a 7∶2 mixture of acetone and methanol was added to the cell suspension and vortexed thoroughly in the dark. Samples were centrifuged for 10 minutes at 10000 g. Absorbance of the supernatant was measured at 775 nm and total bacteriochlorophyll was determined as follows: Abs_775_ * total volume of sample (5 mL) * (bacteriochlorophyll molecular weight (914 g/mol)/bacteriochlorophyll millimolar extinction coefficient (75 mM^−1^ cm^−1^)). Total bacteriochlorophyll in each sample was normalized to total protein content of samples determined using the Lowry assay [77].

### Construction of mutants and expression plasmids

All mutants constructed for this study contained in-frame markerless deletions, which were constructed as previously described [11], [78]. Briefly, regions spanning ∼1500 bp upstream and downstream of the target gene were amplified using sequence-specific primers containing restriction sites for EcoRI, XbaI or HindIII. These fragments were digested with the appropriate restriction enzymes and ligated into pK18mobsacB [79], which had been digested with EcoRI and HindIII, by three-way ligation to generate the various gene deletion constructs, which were confirmed by sequencing. The pK18mobsacB-based plasmids were separately mobilized from *E. coli* S17-1 into relevant *R. sphaeroides* strains. Cells in which the plasmid had successfully integrated into the genome via homologous recombination were identified by selection on SMM plates supplemented with kanamycin. These cells were then grown overnight in SMM without kanamycin [11]. Cells that had lost the *sacB* gene via a second recombination event were identified by growth on SMM plates supplemented with 10% sucrose [11]. Gene deletions were confirmed by PCR and sequencing with specific primers (S8 Table).

To construct plasmids for the ectopic expression of 3x Myc tagged proteins, we modified pIND5 [80] to include 3 copies of a codon optimized Myc tag (EQKLISEEDL – GAGCAGAAGCTGATCTC**G**GAGGAGGACCTG) within the plasmid's multiple cloning site. New multiple cloning sites were added to allowing tagging of proteins either C-terminally (NdeI-PstI-NcoI) or N-terminally (BamHI-SalI-BglII-HindIII). Individual expression plasmids were made by amplifying the target genes from the genome using sequence specific primers (S8 Table) containing restriction sites for NdeI and BglII, HindIII or BamHI for cloning into pIND5 [11] and NdeI/NcoI or BamHI/HindIII for cloning into pIND5-3xMyc. These DNA fragments were digested with the appropriate enzymes and cloned into pIND5 or pIND5-3xMyc digested with the same enzymes. These plasmids were conjugated from *E. coli* S17-1 into the relevant *R. sphaeroides* strains. Cells which harbor the desired plasmid were identified by selection on SMM plates supplemented with kanamycin [11].

### Construction of *lacZ* reporter promoter fusions and β-galactosidase assays

To assay the activity of FnrL and CrpK *in vivo*, β-galactosidase assays were conducted, as previously described [78], [81], in *ΔfnrLΔcrpK* deletion strains containing different promoter-*lacZ* fusions integrated into the genome. To construct these reporter strains, ∼200–300 bp regions upstream of putative target genes (RSP_0281 (*bchE*), RSP_0696 (*ccoN*), RSP_0697 (*usp*), RSP_2346 and RSP_3341), were amplified from genomic DNA using specific primers having NcoI and XbaI restriction sites at their ends (S8 Table). The amplified DNA fragments, as well as a pSUP202 suicide vector containing a promoterless *lacZ* gene [78], were digested with NcoI-XbaI. DNA fragments containing the upstream regulatory sequences were cloned into pSUP202. These promoter-*lacZ* fusion plasmids were then individually conjugated into the *ΔfnrLΔcrpK* strain, generating single copy promoter-*lacZ* fusions integrated in the genome after selecting for the plasmid-encoded tetracycline resistance activity. The *fnrL* and *crpK* genes cloned into pIND5 were conjugated into individual reporter strains and cells harboring the reporter construct and the ectopic expression plasmid were identified by selection with tetracycline and kanamycin. These strains were grown aerobically by shaking 10 mL of culture in 125 mL conical flasks until exponential phase, then were treated with 100 µM IPTG for 3 hrs to increase expression of the indicated TF before measuring β-galactosidase activity as previously described [81].

To assess the contribution of specific bases to FnrL and CrpK activity, β-galactosidase assays were conducted in *ΔfnrLΔcrpK* double deletion strains containing reporter gene fusions of the RSP_0281 (*bchE*) upstream regulatory region with individual point mutations (see Results). These reporter strains were constructed as described above, with individual point mutations being generated by overlap extension PCR. β-galactosidase assays were conducted as described above [11], [78], [81].

### RNA extraction, qRT-PCR and microarray analyses

RNA was isolated from exponential phase cultures of *R. sphaeroides* strains that were grown photosynthetically in 16 mL screw cap tubes or 500 ml cultures in roux bottles with bubbling (95% N_2_, 5% CO_2_) [11],[78]. RNA isolation and subsequent cDNA synthesis, labeling and hybridization to *R. sphaeroides* GeneChip microarrays (Affymetrix, Santa Clara, CA) were performed as previously described [82]. Microarray datasets were normalized by Robust Multichip Average (RMA) to log_2_ scale with background adjustment and quantile normalization [83]. Statistical analysis of normalized data to identify differentially expressed genes was done using the limma package [84]. Correction for multiple testing was done using Benjamini-Hochberg correction [85]. All analyses were conducted in the R statistical programming environment (http://www.R-project.org).

### Chromatin immunoprecipitation analysis (ChIP-qPCR and ChIP-seq analysis)

*R. sphaeroides* cells were grown photosynthetically in 500 ml cultures (see above). For FnrL studies, 3 independent ChIP-seq experiments were conducted for WT cells grown photosynthetically with succinate (2 replicates) or acetate as sole carbon source. For tagged TFs, plasmids expressing the tagged variant of the gene from an IPTG inducible promoter, were cloned into the appropriate mutants (S8 Table). Protein expression was induced with IPTG concentrations (MppG (5 µM), PrrA (10 µM) and CrpK (10 µM)), which restored WT-like growth or pigmentation phenotypes. Cells were harvested at mid-exponential phase and chromatin immunoprecipitation was conducted as previously described [86], using polyclonal antibody against FnrL [24] or against the Myc epitope tag (ab9132, Abcam plc) for all other TFs analyzed. Immunoprecipitated DNA samples were PCR-amplified, gel purified (size selection ∼200 bp) and sequenced at the UW Biotechnoloy Center using the HiSeq 2000 sequencing system (Illumina, Inc). The initial 100 bp sequence tags were trimmed to 70 bp, to remove less reliable DNA sequences, and mapped to the *R. sphaeroides* strain 2.4.1 genome (ftp://ftp.ncbi.nih.gov/genomes/Bacteria/Rhodobacter_sphaeroides_2_4_1_uid57653/) using SOAP version 2.21 [87], allowing a maximum of 3 mismatches and no gaps. Peaks that represent potential TF binding sites were identified using MOSAiCS [88] at a false discovery rate of 0.05. The MOSAiCS analysis was conducted as a two-sample analysis, with control ChIP-seq data generated from Δ*fnrL* grown on acetate (for FnrL analysis), myc antibody ChIP in WT cells (for myc-tagged proteins) or input DNA. Only peaks that were called as significant using both input DNA and an appropriate ChIP control were considered as true peaks. Motifs were identified from within peak regions using MEME [89].

### Accession numbers

All microarray and ChIP-seq datasets generated for this study have been deposited in GEO under the accession GSE58717.

## Supporting Information

S1 Figure Growth of *R. sphaeroides* WT and *ΔfnrL* cells. A comparison of the growth of WT and *ΔfnrL* cells on succinate and acetate.
(PDF)

S2 Figure Difference in growth between PrrA2, PrrA3 and WT cells. (A) A comparison of the growth rates of PrrA2 used in (Eraso et al. 2008), PrrA3 (from this study) and wild type (WT) *R. sphaeroides* cells under anaerobic respiratory conditions (with DMSO), highlighting the significantly faster growth rate of PrrA2. (B) Complementation of PrrA3 with a 3X myc tagged variant of PrrA under photosynthetic conditions. PrrA 3X myc is able to restore photosynthetic growth to PrrA3. The longer lag time of the PrrA3+pIND5-*prrA*-3xmyc relative to WT likely results from the fact that PrrA is already active in the aerobically grown starter culture for the WT cells, but is absent in the complemented strain, at the time of inoculation. This allows WT cells to make a faster transition from aerobic to photosynthetic growth.
(PDF)

S3 Figure Analysis of CrpK. (A) Growth curves comparing photosynthetic growth on succinate of wild type (WT), *ΔfnrL*, *ΔfnrL* cells overexpressing CrpK and a 3X myc tagged variant of CrpK. CrpK and CrpK 3X myc are able to restore photosynthetic growth to *ΔfnrL*. (B) Comparison of the growth of WT and *ΔcrpK* cells on succinate. Deletion of *crpK* has no effect on photosynthetic growth under these conditions. (C) Comparison of the growth of *ΔfnrL* and *ΔfnrL* cells overexpressing CrpK cells on acetate. (D) qPCR analysis of CrpK and FnrL binding at shared (RSP_0697 and *bchE*), FnrL unique (*fnrL*, *hemN*) and CrpK unique (*fliC* and RSP_3604) sites. (E) Comparison of enrichment at shared and unique CrpK and FnrL binding sites between strains expressing either both CrpK and FnrL (WT and *ΔcrpK+crpK*); only FnrL (*ΔcrpK*) or only CrpK (*ΔfnrL-ΔcrpK+crpK*). No significant differences in enrichment for FnrL and CrpK was observed between the strains except at the RSP_0697 promoter, suggest some level of competitive binding might occur here, under physiologically relevant conditions.
(PDF)

S4 Figure Some shared and unique binding sites for FnrL and CrpK. ChIP-seq peaks for select target sites bound by both FnrL and CrpK (RSP_0820, RSP_0166 and *ccoN*), only FnrL *(dorS*, RSP_3341 and *fnrL*) or only CrpK (*fliC*, RSP_3640 and RSP_2349).
(PDF)

S5 Figure Growth curves for *mppG* deletion and over-expression strains. (A) Growth of WT and ΔMppG+pIND5*-mppG* strains aerobically. Over expression of MppG using 50 µM IPTG did not affect aerobic growth of *R. sphaeroides*. (B) Growth of WT and ΔMppG+pIND5*-mppG-*3X myc strains photosynthetically. Over expression of 3X myc tagged MppG using 50 µM IPTG resulted in significant reduction of growth similar to the phenotype observed with the untagged protein.
(PDF)

S1 Table FnrL target genes identified by ChIP-seq analysis of *R. sphaeroides* cells grown photosynthetically.
(XLS)

S2 Table Comparison of gene expression between WT and ΔFnrL during growth on acetate based medium.
(XLSX)

S3 Table Comparison of gene expression between wild type and PrrA3 during anaerobic respiratory growth.
(XLSX)

S4 Table PrrA binding sites identified by ChIP-seq analysis which did not meet criteria used for selecting direct PrrA targets.
(XLSX)

S5 Table Differentially expressed genes between WT and ΔMppG during photosynthetic growth.
(XLSX)

S6 Table Differentially expressed genes between ΔMppG*+*pIND5-*mppG* and ΔMppG during photosynthetic growth.
(XLSX)

S7 Table MppG binding sites identified by ChIP-seq analysis which did not meet criteria used for selecting direct MppG targets.
(XLSX)

S8 Table Plasmids, strains and primers used in this study.
(XLSX)
