# Supplementary figures and images for: Global Analysis of Photosynthesis Transcriptional Regulatory Networks
Source: PLoS Genet. 2014 Dec 11;10(12):e1004837. doi: 10.1371/journal.pgen.1004837 (PMC4263372; doi:10.1371/journal.pgen.1004837)

## Succinate

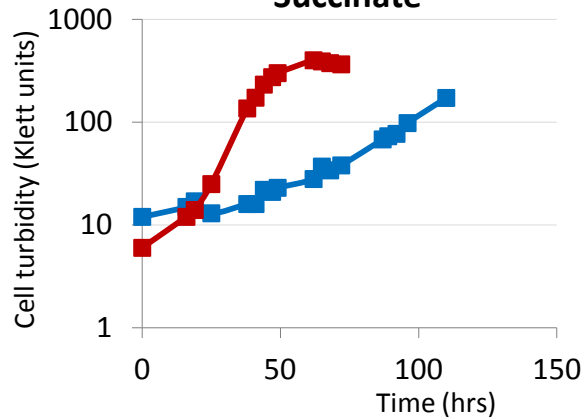

WT

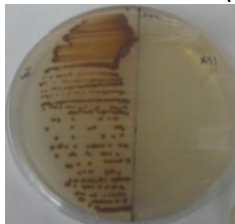

$\Delta fnrL$

## Acetate

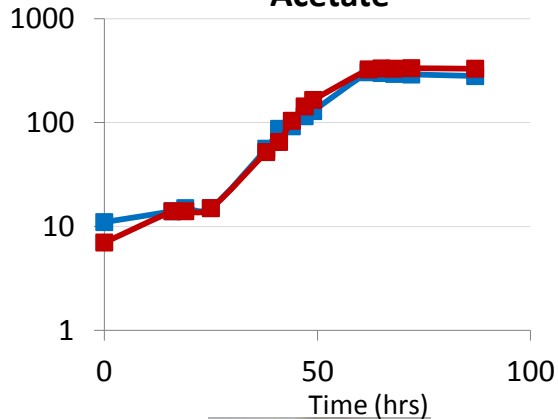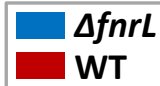

WT

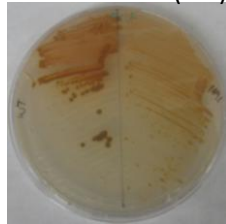

$\Delta fnrL$

Supplement: S1 Figure — Growth of R. sphaeroides WT and ΔfnrL cells. A comparison of the growth of WT and ΔfnrL cells on succinate and acetate. (PDF) [file pgen.1004837.s001.pdf]

A.

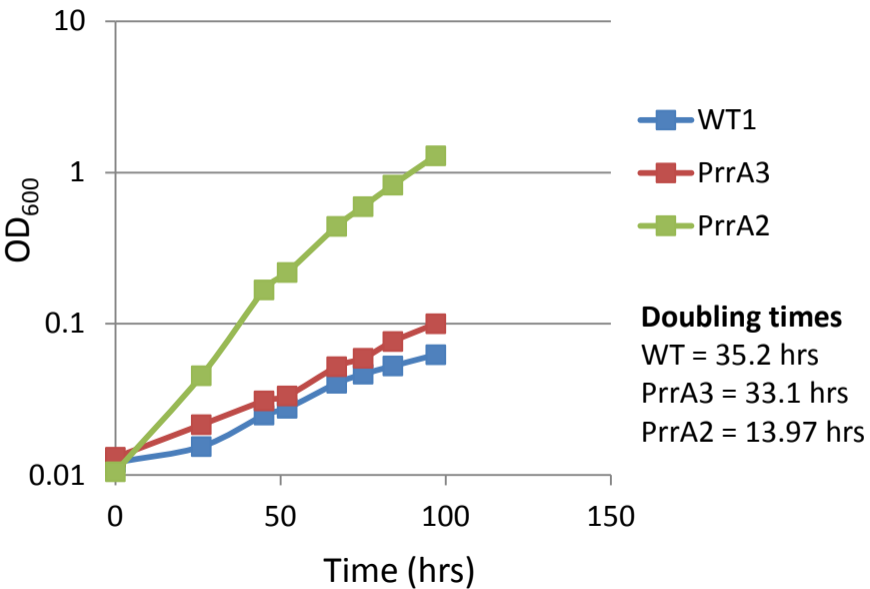

B.

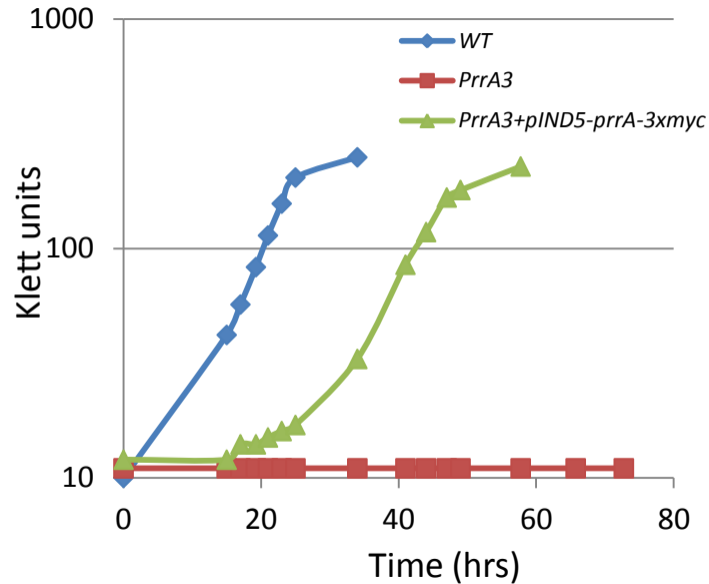

Supplement: S2 Figure — Difference in growth between PrrA2, PrrA3 and WT cells. (A) A comparison of the growth rates of PrrA2 used in (Eraso et al. 2008), PrrA3 (from this study) and wild type (WT) R. sphaeroides cells under anaerobic respiratory conditions (with DMSO), highlighting the significantly faster growth rate of PrrA2. (B) Complementation of PrrA3 with a 3X myc tagged variant of PrrA under photosynthetic conditions. PrrA 3X myc is able to restore photosynthetic growth to PrrA3. The longer lag time of the PrrA3+pIND5-prrA-3xmyc relative to WT likely results from the fact that PrrA is already active in the aerobically grown starter culture for the WT cells, but is absent in the complemented strain, at the time of inoculation. This allows WT cells to make a faster transition from aerobic to photosynthetic growth. (PDF) [file pgen.1004837.s002.pdf]

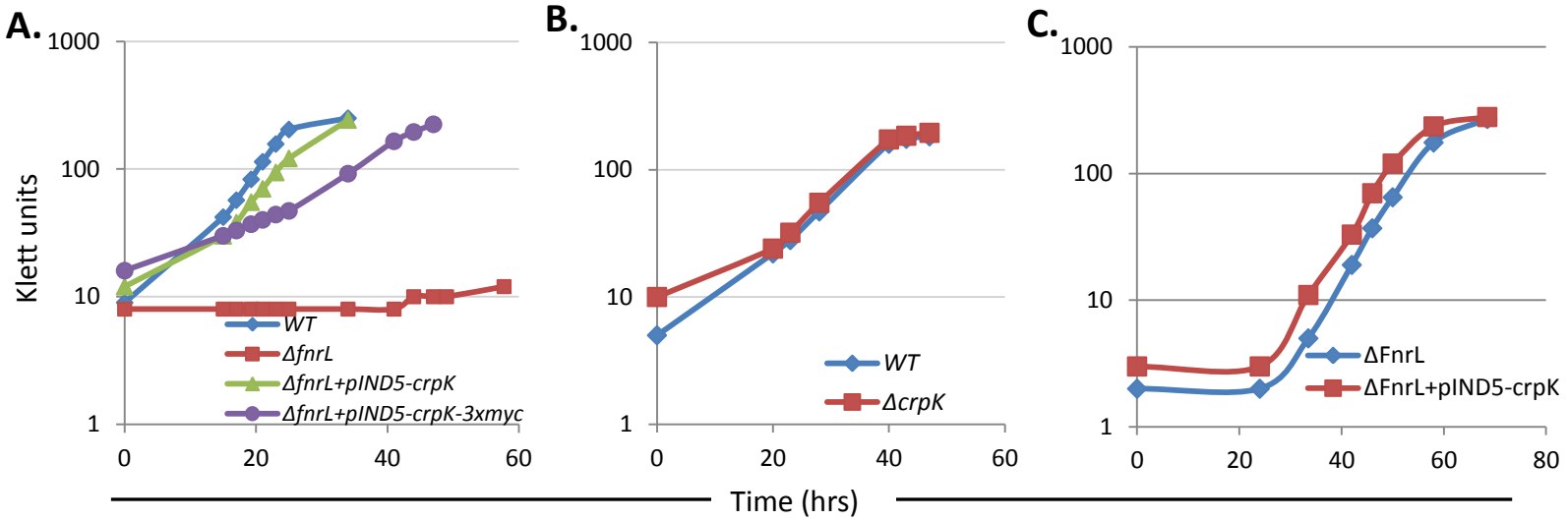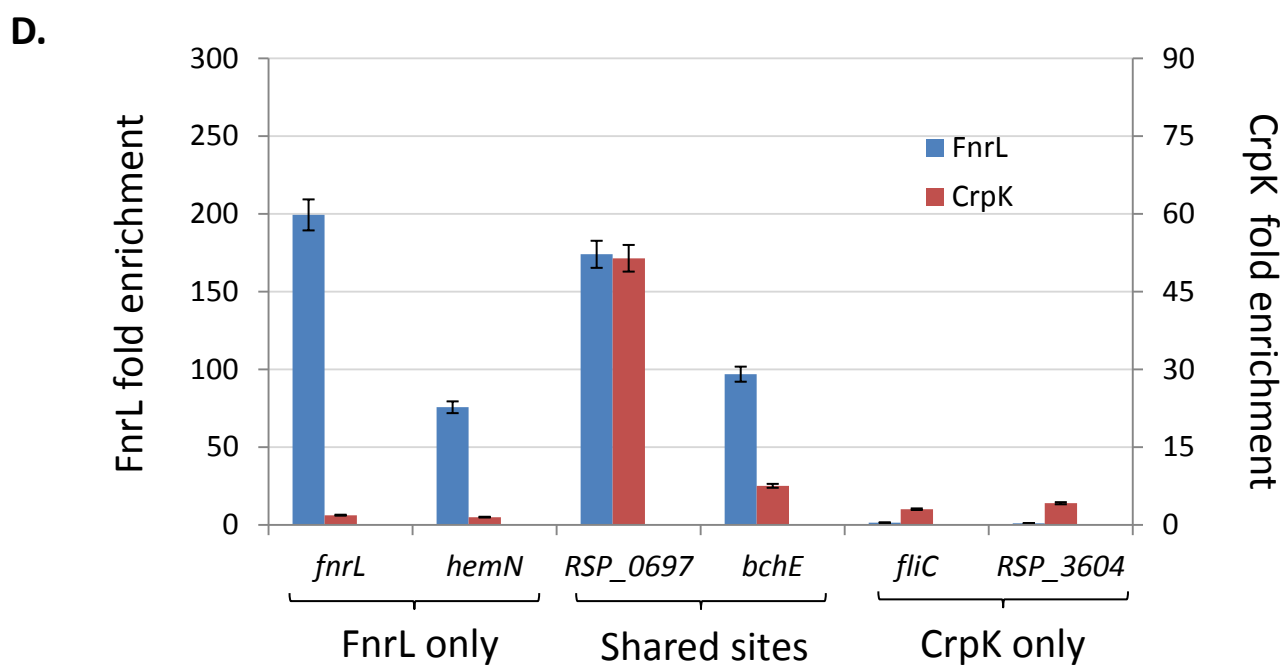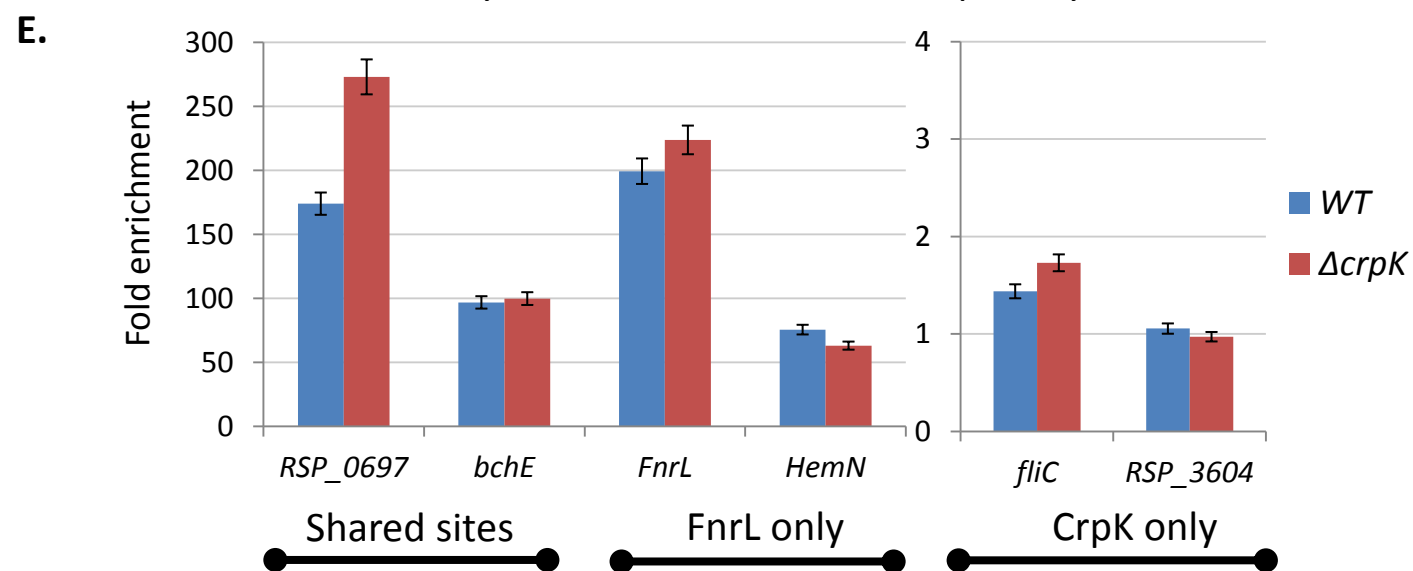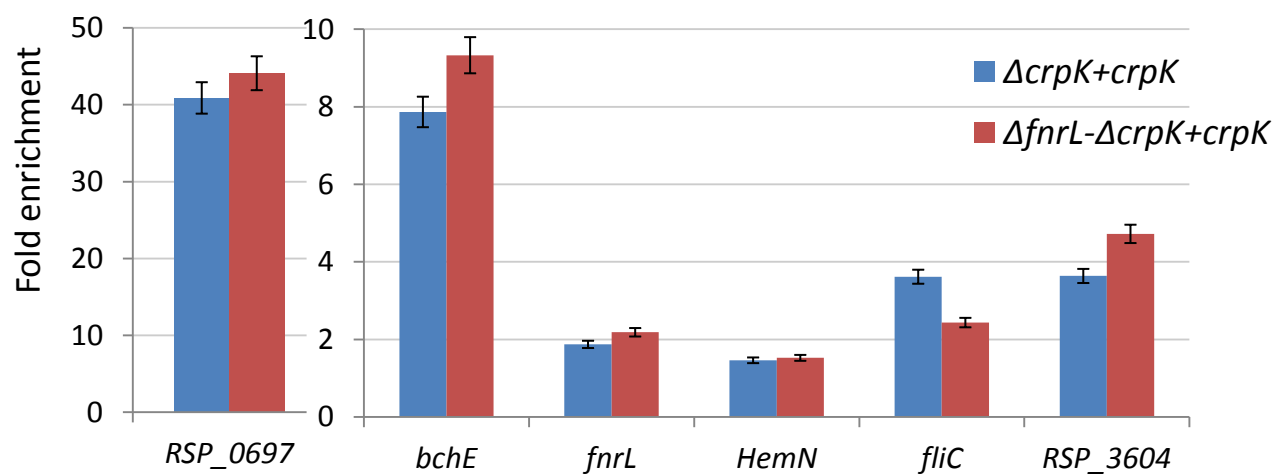

Supplement: S3 Figure — Analysis of CrpK. (A) Growth curves comparing photosynthetic growth on succinate of wild type (WT), ΔfnrL, ΔfnrL cells overexpressing CrpK and a 3X myc tagged variant of CrpK. CrpK and CrpK 3X myc are able to restore photosynthetic growth to ΔfnrL. (B) Comparison of the growth of WT and ΔcrpK cells on succinate. Deletion of crpK has no effect on photosynthetic growth under these conditions. (C) Comparison of the growth of ΔfnrL and ΔfnrL cells overexpressing CrpK cells on acetate. (D) qPCR analysis of CrpK and FnrL binding at shared (RSP_0697 and bchE), FnrL unique (fnrL, hemN) and CrpK unique (fliC and RSP_3604) sites. (E) Comparison of enrichment at shared and unique CrpK and FnrL binding sites between strains expressing either both CrpK and FnrL (WT and ΔcrpK+crpK); only FnrL (ΔcrpK) or only CrpK (ΔfnrL-ΔcrpK+crpK). No significant differences in enrichment for FnrL and CrpK was observed between the strains except at the RSP_0697 promoter, suggest some level of competitive binding might occur here, under physiologically relevant conditions. (PDF) [file pgen.1004837.s003.pdf]

# Targets bound by both FnrL and CrpK

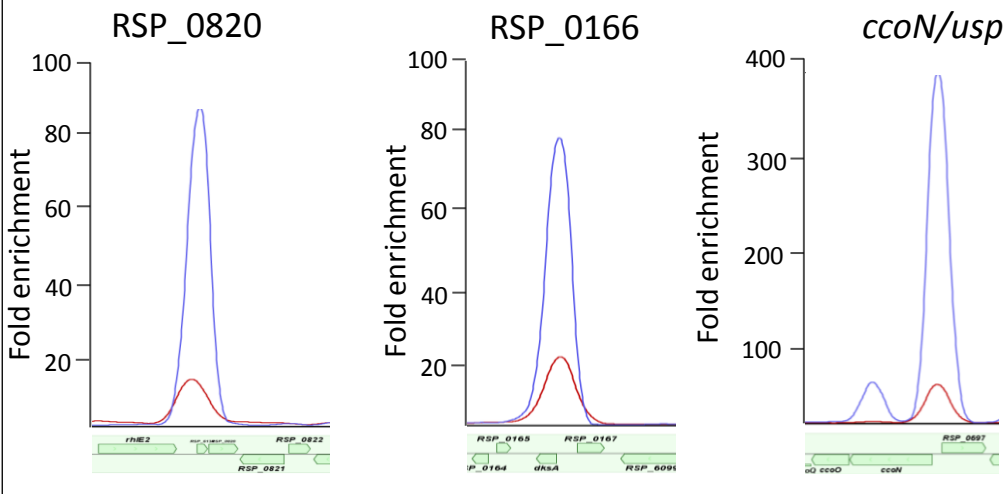

## Targets bound by only FnrL

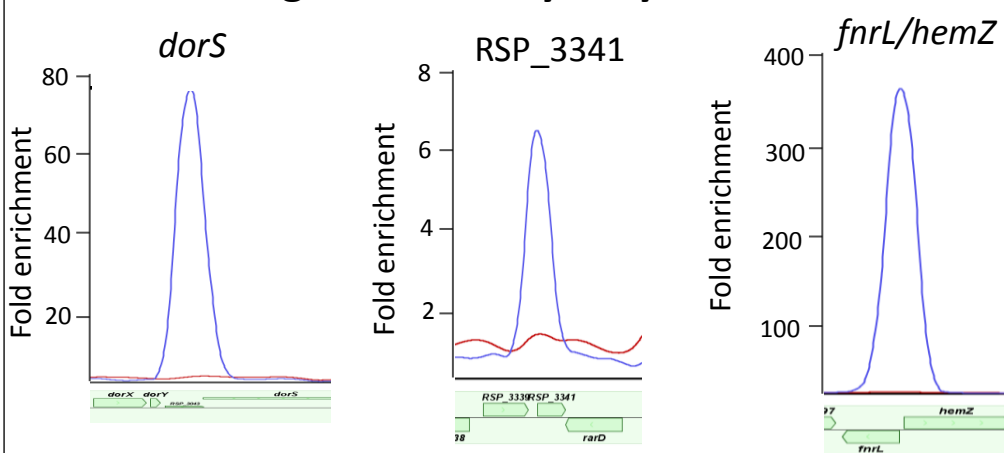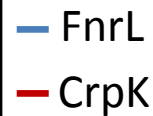

## Targets bound by only CrpK

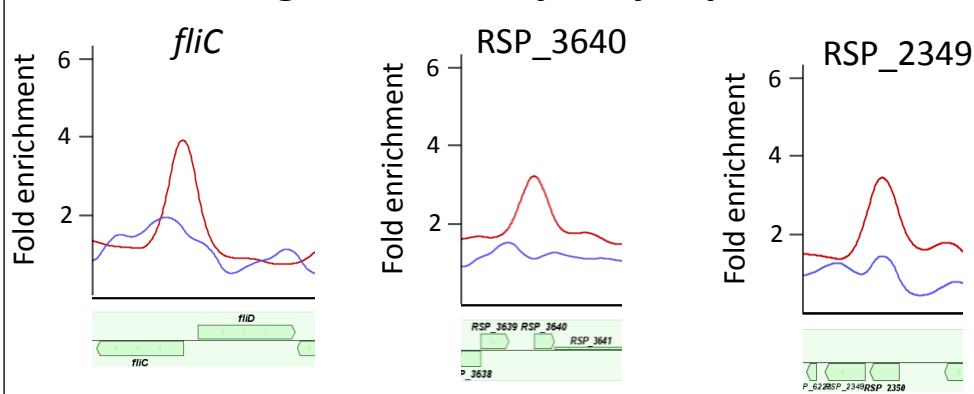

Supplement: S4 Figure — Some shared and unique binding sites for FnrL and CrpK. ChIP-seq peaks for select target sites bound by both FnrL and CrpK (RSP_0820, RSP_0166 and ccoN), only FnrL (dorS, RSP_3341 and fnrL) or only CrpK (fliC, RSP_3640 and RSP_2349). (PDF) [file pgen.1004837.s004.pdf]

A.

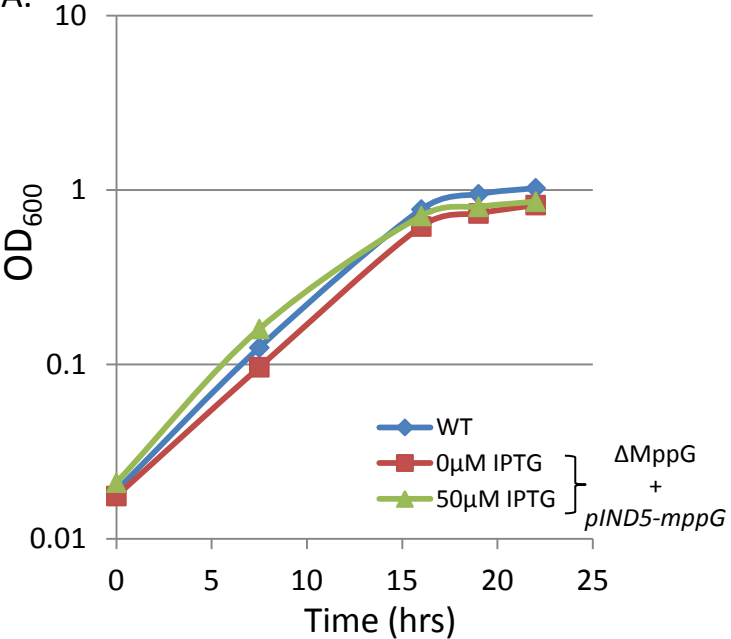

B.

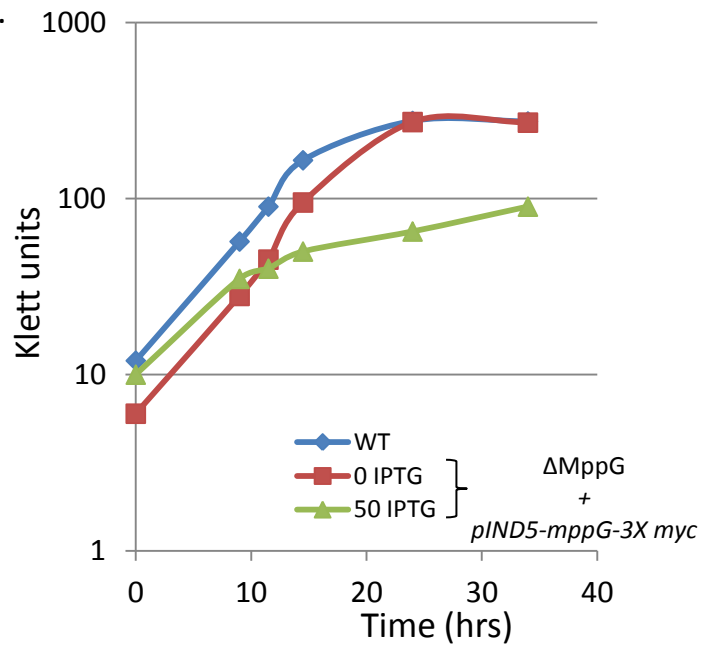

Supplement: S5 Figure — Growth curves for mppG deletion and over-expression strains. (A) Growth of WT and ΔMppG+pIND5-mppG strains aerobically. Over expression of MppG using 50 µM IPTG did not affect aerobic growth of R. sphaeroides. (B) Growth of WT and ΔMppG+pIND5-mppG-3X myc strains photosynthetically. Over expression of 3X myc tagged MppG using 50 µM IPTG resulted in significant reduction of growth similar to the phenotype observed with the untagged protein. (PDF) [file pgen.1004837.s005.pdf]
